# Simultaneous high-speed imaging and optogenetic inhibition in the intact mouse brain

**DOI:** 10.1038/srep40041

**Published:** 2017-01-05

**Authors:** Serena Bovetti, Claudio Moretti, Stefano Zucca, Marco Dal Maschio, Paolo Bonifazi, Tommaso Fellin

**Affiliations:** 1 Optical Approaches to Brain Function Laboratory, Department of Neuroscience and Brain Technologies, Istituto Italiano di Tecnologia, Via Morego 30, 16163 Genova, Italy; 2 School of Physics and Astronomy, Italy-Israel Joint Neuroscience Laboratory, Tel Aviv University, 69978 Tel Aviv, Israel; 3 Computational Neuroimaging Lab, BioCruces Health Research Institute, Plaza de Cruces, s/n E-48903, Barakaldo, Spain

## Abstract

Genetically encoded calcium indicators and optogenetic actuators can report and manipulate the activity of specific neuronal populations. However, applying imaging and optogenetics simultaneously has been difficult to establish in the mammalian brain, even though combining the techniques would provide a powerful approach to reveal the functional organization of neural circuits. Here, we developed a technique based on patterned two-photon illumination to allow fast scanless imaging of GCaMP6 signals in the intact mouse brain at the same time as single-photon optogenetic inhibition with Archaerhodopsin. Using combined imaging and electrophysiological recording, we demonstrate that single and short bursts of action potentials in pyramidal neurons can be detected in the scanless modality at acquisition frequencies up to 1 kHz. Moreover, we demonstrate that our system strongly reduces the artifacts in the fluorescence detection that are induced by single-photon optogenetic illumination. Finally, we validated our technique investigating the role of parvalbumin-positive (PV) interneurons in the control of spontaneous cortical dynamics. Monitoring the activity of cellular populations on a precise spatiotemporal scale while manipulating neuronal activity with optogenetics provides a powerful tool to causally elucidate the cellular mechanisms underlying circuit function in the intact mammalian brain.

Mapping the activity of neuronal networks in space and time *in vivo* is crucial to advance our understanding of the brain. However, this aim poses distinct challenges because it requires: *i)* attaining high spatial resolution (cellular processes can be thinner than 1 μm); *ii)* monitoring reasonably large fields of view (neuronal networks can extend over hundreds of microns); *iii)* recording cellular activity with high temporal resolution (electrical signals in the brain can be on the scale of milliseconds); and *iv)* discriminating between the different cell types that are present in the brain. Moreover, techniques for mapping neuronal activity should ideally be compatible with optogenetics to allow causal control of electrical activity in identified cellular subtypes. Two-photon fluorescence imaging is recognized as a promising approach to achieve most of these requirements^1^,^2^. Indeed, laser-scanning two-photon microscopy (LSTPM) has been used to monitor cellular activity in the intact mammalian brain with near diffraction-limited spatial resolution over hundreds of microns^3^,^4^,^5^, and a variety of approaches have been developed to increase the temporal resolution of imaging^6^,^7^,^8^,^9^,^10^. Moreover, genetically encoded fluorescent calcium indicators^11^,^12^,^13^ can be expressed in a cell-type specific manner to monitor neural activity, and the latest versions of these indicators have kinetics comparable to those of the fastest synthetic dyes^14^. Ideally, pairing fast imaging with simultaneous single-photon optogenetics would provide a powerful experimental approach^15^, combining the fine resolution of two-photon microscopy with the ability to reveal causal interactions between neuronal populations. However, when imaging is performed over multiple cells fast two-photon imaging may suffer from limited signal-to-noise ratio (SNR) because at constant acquisition speed the effective dwell time *per* individual cell decreases with the number of imaged cells^9^,^10^. These weak signals are usually detected by highly sensitive photomultiplier tubes (PMTs) and, when fast imaging is coupled with single-photon optogenetics, the light intensities commonly used for single-photon opsin activation generate large artifacts in the calcium signals acquired by PMTs^16^,^17^, requiring a blanking period and ultimately preventing simultaneous fast imaging and optogenetic manipulation. *Simultaneous* imaging is especially crucial for optogenetic silencing experiments because the effects of inhibitory opsin activation on neuronal circuits are optimally observed *during* the optical stimulation.

Here, we developed a two-photon microscope for effective high-speed imaging of GCaMP6 indicators^14^,^18^ during parallel wide-field single-photon excitation of the inhibitory opsin archaerhodopsin (Arch)^19^ in the intact mouse brain *in vivo*. The optical system is based on phase modulation of the excitation light^20^,^21^,^22^,^23^,^24^ and performs imaging with a camera by simultaneously projecting near diffraction-limited stationary laser spots only onto cells expressing the calcium indicator (scanless imaging).

## Results

**Figure 1:**
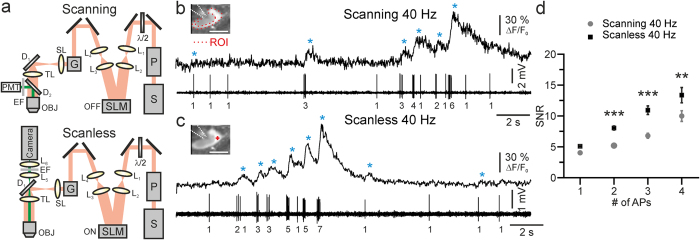
Higher SNR of GCaMP signals in scanless than scanning imaging. (**a)** Set-up for scanning (top) and scanless (bottom) imaging. S, laser source; P, Pockels cell; λ/2, half-wave plate; L_1–6_ lenses; SLM, spatial light modulator; G, galvanometric mirrors; SL, TL, scan and tube lenses; D_1_, D_2_, dichroic mirrors; EF, emission filters; PMT, photomultiplier tube; OBJ, objective. (**b**) Simultaneous scanning imaging (top trace) and juxtasomal recording (bottom trace) from a GCaMP6s-expressing layer II/III neuron (inset) in an anesthetized mouse. Number of discharged APs is indicated below the electrophysiological trace. The blue asterisks indicate calcium events that are detected in the analysis (see Methods). The red dotted line in the inset represents the borders of the ROI used to calculate the fluorescence signal. Inset: scale bar, 10 μm. (**c**) Simultaneous scanless imaging (top trace, illumination with 1 point represented by the red cross in inset, power value: 20 mW) and juxtasomal recording (bottom trace) from the same cell shown in (**b**) in which scanning imaging was performed. Depth: 80 μm. Inset: scale bar, 10 μm. (**d**) SNR as a function of the number of APs in the two recording modes. Two-way ANOVA, p = 8E-14 for SNR *vs* imaging modality, p < 1E-15 for SNR *vs* AP number, interaction p = 0.018; 40–106 events from 10 cells. In this as well as in other figures: *p < 0.05; **p < 0.01; ***p < 0.001.

The microscope is based on a commercial liquid crystal spatial light modulator (SLM) and a galvanometric mirror-based laser scan head (Fig. 1a). Emitted fluorescence was collected either in non-descanned mode by a PMT (Fig. 1a, top) or with a camera (Fig. 1a, bottom). Two main steps were needed in our experimental approach. First, a high-resolution image of the sample was acquired in the laser-scanning configuration, using the galvanometric mirrors in combination with the PMT (*scanning*, Fig. 1a top). Based on this image, multiple regions of interest (ROIs) in the sample were identified, and custom software was used to compute a proper phase map to control the SLM (see Methods). Phase modulation in the plane optically conjugated to the objective back-focal plane produced illumination of the sample only in the desired ROIs. In this second step (*scanless,* Fig. 1a bottom), the galvanometric mirrors were held stationary, and emitted fluorescence photons were detected simultaneously across all the ROIs by a camera. Switching between the two configurations required only moving the dichroic mirror D_2_ (Fig. 1a). In the scanless mode, the dwell time is independent of *N* (Supplementary Fig. S1); it is equal to the inverse of the acquisition frequency and is thus significantly longer that in the scanning mode (Supplementary Fig. S1).

### Scanless fluorescence imaging of GCaMP indicators in the intact mouse brain

We initially tested our optical set-up by performing scanning and scanless functional imaging of fluorescence signals at the same acquisition frequency (40 Hz) in anesthetized mice expressing the genetically encoded calcium indicator GCaMP6s in layer II/III cortical neurons in combination with juxtasomal electrophysiological recordings (Fig. 1b,c). Scanning and scanless imaging was performed sequentially on each electrophysiologically recorded cell, so that fluorescence signals generated in the same neuron could be recorded in the two imaging configurations and appropriately compared. Correlated fluorescence transients and electrophysiological signals corresponding to single action potentials (APs) and trains of APs were detected in both imaging modalities. However, calcium events showed significantly higher SNRs for trains of APs in scanless compared to scanning imaging (Fig. 1d). We then extended these findings comparing fast scanless imaging with fast linescan imaging (Supplementary Fig. S2). In both recording configurations the acquisition frequency was 1 kHz and only one cell was imaged in each imaging modality. Similarly to what observed before, we found that calcium events recorded in the scanless modality tended to have higher SNR for trains of APs compared to events recorded in the fast line scan configuration (Supplementary Fig. S2). In interpreting these results it is important to consider that they depend on the characteristics of the fluorescence detectors used in scanning and scanless imaging, a multialkali PMT (Hamamatsu, Milan, Italy) and NeuroCCD-SMQ camera (Redshirt Imaging, Decatur, GA), respectively.

We next examined whether the scanless approach induced appreciable probe photobleaching and tissue photodamage (Supplementary Fig. S3). Photobleaching of GCaMP6 was first assessed by illuminating layer II/III neurons *in vivo* in the scanless modality over a time window of >30 s. To induce a reliable and rhythmic calcium signal, we boosted spontaneous activity in the cortex by applying the GABA_A_ receptor antagonist gabazine (10 μM). To increase the probability of inducing photobleaching and thus perform a stricter control experiment, we illuminated each cell with more than one point (# of points per cell: 2–4). Given that we kept the power per point constant (20 mW per point), under this experimental condition, we delivered to the imaged cell an average of 2–4 times the power that we used in other scanless population imaging experiments throughout this study (in which only one point per cell was used for scanless imaging). Importantly, even under these experimental conditions, no major sign of bleaching in GCaMP6-expressing neurons was observed (Supplementary Fig. S3a,b). Average values of baseline change were: −0.1 ± 0.1% for illumination of a cell with two points; 0.3 ± 0.1% for illumination with 4 points. Student’s *t*-test, p = 0.028, N = 11 cells from 3 animals. We controlled for potential induction of photodamage by comparing cell morphology and calcium dynamics (recorded using the microscope in the scanning configuration) before and after a five minute-long scanless imaging session. We could not detect any change in the morphology of the imaged cells (Supplementary Fig. S3c–f), nor did we detect any significant modification of their calcium dynamics (Supplementary Fig. S3g,h). We further tested for potential photodamage effects by monitoring the frequency of APs using juxtasomal recordings on GCaMP6-positive cells during prolonged (up to 5 minutes) scanless illumination. No significant changes in the frequency of APs were detected in the first 30 s with respect to the last 30 s of the scanless imaging session (average firing rate in the first 30 s: 1.58 ± 0.21 Hz; average firing rate in the last 30 s: 1.59 ± 0.19, paired Wilcoxon test, p = 0.75, N = 20 cells). We then extended these results to longer time windows (up to one hour) and compared the effects of prolonged imaging on recorded calcium signals in the scanless and scanning configurations (3 minute-long imaging sessions, 1 imaging session every 10 minutes for both scanning and scanless imaging). No significant change in the SNR of calcium events was observed in imaged cells over time in either imaging configuration (Supplementary Fig. S3i). Multiple scanless imaging sessions also did not result in appreciable morphological changes at the cortical surface (Supplementary Fig. S3j,k).

**Figure 2:**
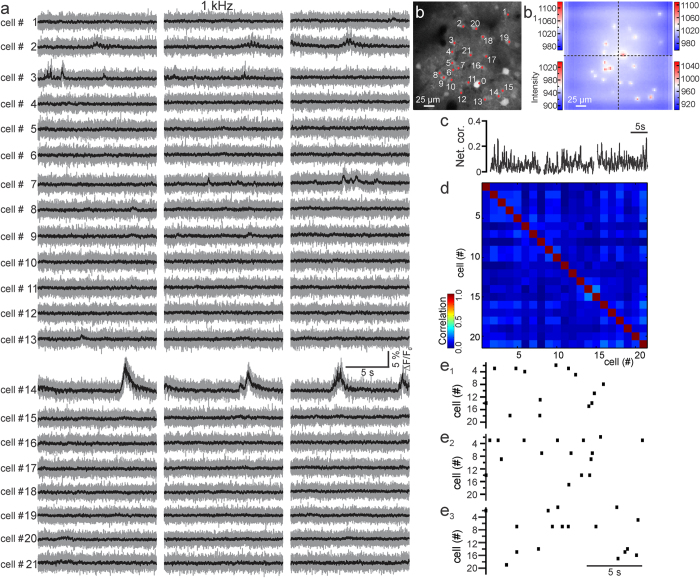
Fast scanless mapping of sparse cortical dynamics *in vivo*. (**a**) Fluorescence signals over time from 21 cortical neurons, imaged in the scanless configuration at 1 kHz in an anesthetized mouse. λ = 920 nm; power, <13 mW per spot. Depth: 140 μm. ΔF/F_0_ are shown in grey; black lines represent a posteriori filtered traces (τ = 15 ms, see Methods). (**b**) Scanning image showing the GCaMP6f-expressing neurons imaged in (**a**). The red crosses indicate the positions of the spots used for scanless imaging. Each cell is identified with a number in (**b**). Cell number 0 was not plotted in (**a**) and used only as spatial reference. (**b**_**1**_) Fluorescence signals recorded with the camera during scanless multipoint illumination of the neurons indicated in (**b**). Four pseudocolor scales are shown corresponding to the four camera detector quadrants (see Methods for details). (**c,d**) Instantaneous network correlation as a function of time (**c**) and pair-wise correlation (**d**) for the experiment displayed in (**a**). (**e**_**1**_**–e**_**3**_) Raster plots of the calcium transient onsets (see Methods) for the three recordings shown in (**a**).

**Figure 3:**
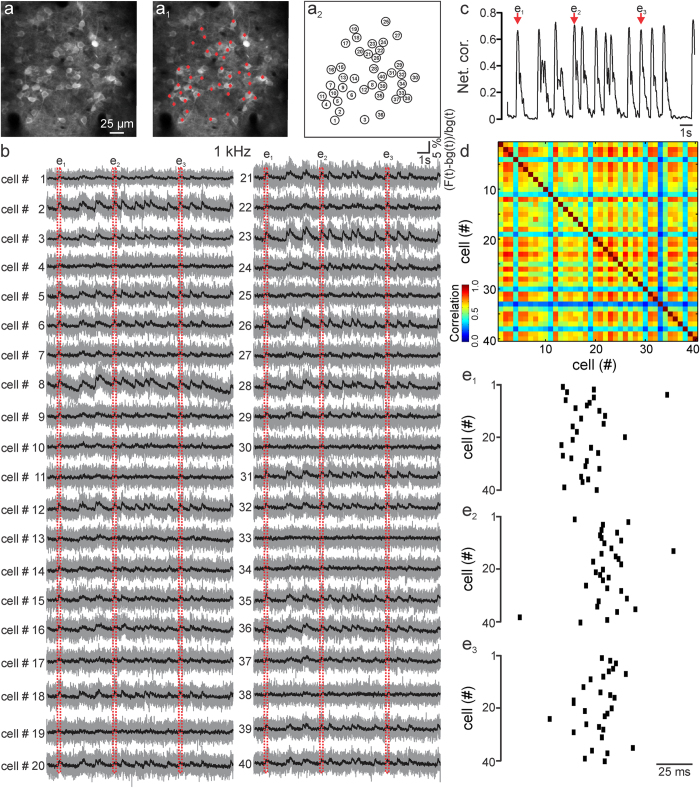
Fast scanless mapping of synchronous cortical activities *in vivo*. (**a–a**_**2**_) Layer II/III neurons expressing GCaMP6f (**a**) in an anesthetized mouse and positions of the light spots that were projected for scanless fluorescence imaging (**a**_**1**_). Cell number is displayed in (**a**_**2**_). Depth: 135 μm. (**b**) Fluorescence signals over time from the 40 cells displayed in (**a**_**1**_**,a**_**2**_), imaged in the scanless configuration at 1 kHz. Spontaneous activity was recorded in the presence of gabazine (10 μM). λ = 920 nm; power, <5.5 mW per spot. Values of (F(t) − bg(t))/bg(t) are shown in grey; black lines represent a posteriori filtered traces (τ = 15 ms, see Methods). (**c,d**) Instantaneous network correlation as a function of time (**c**) and pair-wise correlation (**d**) for the experiment displayed in (**b**). (**e**_**1**_**–e**_**3**_) Raster plots of the calcium transient onsets (see Methods) for the three time windows highlighted (red dotted line) in (**b**).

We characterized the spatial resolution of our system by measuring the point-spread function (PSF) at different lateral positions of the scanless field of view (FOV). Using phase modulation, a single excitation spot (λ_exc_ = 920 nm; microscope objective: LUMPlanFl40X/IR objective, 0.8 NA) was placed over a subresolved fluorescent bead (0.17 μm in diameter), and the PSF was acquired in scanning mode (Supplementary Fig. S4a–c, full-width-half-maximum at radial position 0 μm, FWHM_x,y_ = 0.51 ± 0.03 μm and FWHM_x,z_ = 2.65 ± 0.15 μm, N = 10). The FWHM_x,y_ and FWHM_x,z_ of the PSF remained nearly constant as a function of the bead position in the FOV (Supplementary Fig. S4d), and they were independent of the number of points that were simultaneously projected using the SLM (Supplementary Fig. S4e,f). Because of limited diffraction efficiency, the intensity of fluorescent signals decreased when points were projected in lateral parts of the FOV (Supplementary Fig. S4g). In functional recordings (e.g., Figs 2 and 3), this decrease was partially compensated for via software control of the SLM (see Methods).

Because in the scanless configuration parallel excitation is provided at the sample and the fluorescence is collected by a camera, the scattering of emitted fluorescence photons from one point of illumination may contaminate the signal generated by a neighboring illuminated point (cross talk). To quantify this effect as a function of depth within the tissue, we first recorded the image of a fluorescent bead (1.75 μm in diameter) with the camera in the scanless configuration while interposing cortical slices of different thickness between the bead and the detector (Supplementary Fig. S5). The diameter of the bead was chosen to be similar to the dimensions of the biological structures that we wanted to image *in vivo*. As expected, the FWHM in the x, y and x, z planes of the bead image increased as a function of slice thickness (Supplementary Fig. S5c,d). We then extended these *in vitro* results to anesthetized animals *in vivo* and recorded in the scanless configuration from two GCaMP6s-expressing neurons that were active simultaneously (distance between the cells: ~10 μm) as a function of the neurons’ depth within the tissue (Supplementary Fig. S6). As shown in Supplementary Fig. S6h, the ability to separate two peaks *S* decreases with depth within the tissue. Illuminating only one of the two points at a time, we also found that the contamination *C* increases with tissue depth and stayed <7% for recording depth <250 μm (Supplementary Fig. S6i). In scanless imaging, it is thus possible to discriminate the signals coming from two adjacent and simultaneously active neurons with limited cross talk for cells located within the first 250 μm from the cortical surface.

Because the PSF was more elongated in the axial than in the radial direction (Supplementary Fig. S4a–d), GCaMP6-expressing structures located immediately above or below the target cell might be sources of contamination. Using simulation based on experimental measures of PSF, and cell dimension, we determined that the neuropil could contribute up to 30% the signal generated at one illuminated point but this only marginally contaminated the signal of a nearby illuminated cell (see Methods and Supplementary Fig. S7). Moreover, we also estimated that a cell positioned above or below and in close contact with another illuminated neuron could contaminate up to 18% of the signal generated at the illumination site. However, based on the cell-to-cell distance distribution measured under our experimental conditions only <4% of the total number of cell pairs are in close contact with one another (see Methods).

### Fast scanless imaging of action potential firing in GCaMP6-expressing cortical networks in the intact mouse brain

We used the scanless configuration to perform high-speed imaging of neuronal networks comprising tens of GCaMP6f-expressing cells in anesthetized (Figs 2 and 3) or awake, head restrained mice (Supplementary Fig. S8) *in vivo*. In anesthetized mice, we imaged spontaneous activity at 1 kHz in networks of 9–47 layer II/III neurons at a depth of 100–160 μm in the cortex, under control conditions (Fig. 2) and in the presence of gabazine (10 μM, Fig. 3). Each cell was illuminated with one spot (<5–17 mW per spot; see Methods for power calculation). Under control conditions (Fig. 2a–c), fluorescence transients recorded in the scanless configuration had an average frequency of 0.038 ± 0.004 Hz (N = 118 cells from 6 experiments in 5 animals). Activity among cells was also characterized by poor temporal correlation (Fig. 2d-e3, average value of network correlation: 0.09 ± 0.02, N = 6 experiments; see Methods). This is consistent with the sparse neuronal activity observed in superficial cortical layers with electrophysiological^25^,^26^,^27^ or LSTPM^28^,^29^ recordings and demonstrates negligible cross talk between neighboring cells in the scanless imaging configuration. When inhibition was blocked by adding gabazine, cells displayed repetitive calcium events (Fig. 3a–c, average event frequency: 0.55 ± 0.02 Hz, N = 145 cells from 6 experiments in 4 animals), with spontaneous activity largely correlated among different cells (Fig. 3d, average value of correlation among cells: 0.51 ± 0.05, N = 6 experiments, significantly higher than that recorded in the absence of gabazine, Student’s *t*-test, p = 1.3E-4). Importantly, despite the fact that events were largely correlated across neurons, each cell displayed a distinct timing of initiation of the calcium event with respect to its neighbors (Fig. 3e_1_– e_3_). Moreover, pairwise correlation and the relative timing of calcium onsets between two cells did not strongly depend on the distance between the two cells in a range of 0–50 μm (Supplementary Fig. S9), further confirming the absence of significant cross talk among cells in superficial cortical layers in the scanless configuration. It is important to note that to minimize signal crosstalk during the correlated network activities induced by gabazine (Fig. 3), we background subtracted fluorescent signals using a spatial filter (see Methods). In this case, the background (*bg*) was calculated in a region surrounding the position of the illuminating spot and fluorescence signals were displayed as (F(t)-bg(t))/bg(t). In contrast, in Fig. 2 where spontaneous activity is sparse, we showed fluorescence signals as (F(t)-F_0_)/F_0_, where F_0_ is the fluorescence signal in the absence of activity in the same pixels where F(t) is calculated.

**Figure 4:**
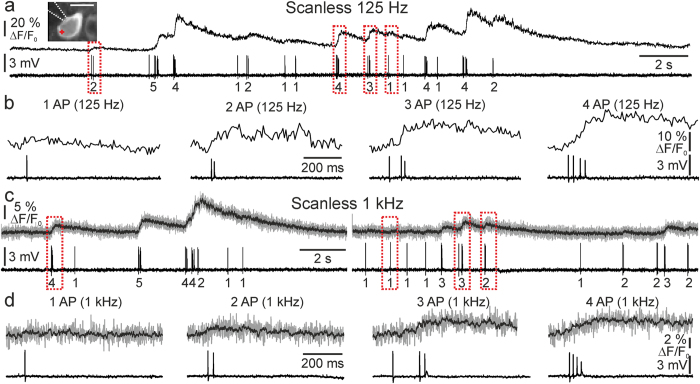
High-speed scanless imaging of single APs and trains of APs *in vivo*. (**a**) Simultaneous scanless imaging (top trace) and juxtasomal electrophysiological recording (bottom trace) from a GCaMP6s-expressing layer II/III neuron (inset) in an anesthetized mouse. Number of discharged APs is indicated below the electrophysiological trace. Illumination with one spot (the red cross in the inset), λ = 920 nm, acquisition frequency 125 Hz, power value: 30 mW. Depth = 75 μm. Inset: scale bar, 10 μm. (**b**) Traces are shown at an enlarged time scale for one (leftmost panel), two (second panel), three (third panel) and four (rightmost panel) APs. The portions of traces shown in (**b**) are indicated in (**a**) by the red dotted line. (**c,d)** Same as in (**a,b**) but at acquisition frequency of 1 kHz. Grey lines, raw ΔF/F_0_ values; black lines, *a posteriori* filtered data (τ = 5 ms, see Methods).

By combining LSTPM and electrophysiological recording, it has been shown that GCaMP indicators, and in particular GCaMP6, are sensitive enough to detect, under favorable circumstances^30^, the calcium elevation elicited by single APs in pyramidal neurons *in vivo*^14^,^31^. In the scanless configuration, we could image GCaMP6 signals at high acquisition frequencies (Fig. 2). To examine in detail the relationship between APs and calcium signals at these high acquisition frequencies, we combined juxtasomal electrophysiological recording and scanless imaging experiments (Fig. 4) in single layer II/III pyramidal neurons expressing GCaMP6s in anesthetized mice. Principal neurons were targeted through combined injection of wild type mice with AAVs carrying a Cre sequence under the CaMKII promoter and AAVs carrying an inverted GCaMP6s sequence flanked between two incompatible loxP variants (double-floxed). At an acquisition frequency of 125 Hz (Fig. 4a,b), the average percentage of calcium events correlated with electrophysiologically recorded APs was 100 ± 0%, measured from 61 calcium events in N = 3 cells. The detection accuracy (see Methods for definition) and the SNR of the calcium events increased with the number of APs within the train (Kruskal-Wallis test, p = 3.5E-3 for detection accuracy and one-way ANOVA, p = 1.8E-6 for SNR). For a single AP, the detection accuracy was 18.3 ± 5.1% (N = 3 cells), and the SNR of *detected* events was 4.1 ± 0.3 (N = 12 events from 3 cells). For AP doublets, the detection accuracy was 78.6 ± 6.0% (N = 3 cells), and the SNR of *detected* events was 7.9 ± 0.6 (N = 18 events from 3 cells). For AP triplets, the detection accuracy was 100.0 ± 0.0% (N = 3 cells), and the SNR was 11.8 ± 2.0 (N = 9 events from 3 cells). For trains of four APs, the detection accuracy was 100.0 ± 0.0% (N = 3 cells), and the SNR was 14.6 ± 1.9 (N = 11 events from 3 cells). The detection accuracy was variable across cells possibly due to the variability of GCaMP6 expression level in different cells. We also measured the SNR of all calcium signals (*detected* and *undetected*) using the electrophysiological recording as ground-truth. The SNR for all single APs was 1.8 ± 0.2 (N = 69 events from 3 cells) and for all AP doublets the SNR was 6.9 ± 0.7 (N = 22 events from 3 cells).

At an acquisition frequency of 1 kHz (Fig. 4c,d), the average percentage of calcium events correlated with electrophysiologically recorded APs was 96 ± 4%, measured from 47 calcium events in N = 6 different cells. The detection accuracy of APs and the SNR of the calcium events increased with the number of APs within the train (Kruskal-Wallis test, p = 0.034 for accuracy and one-way ANOVA, p= 3.7E-4 for SNR). For single APs, detection accuracy was 28.9 ± 16.2% (N = 6 cells), and the SNR of the *detected* calcium transients was 3.0 ± 0.2 (N = 11 events from 4 cells). For AP doublets, detection accuracy was 63 ± 17% (N = 4 cells), and the SNR of the *detected* calcium transients was 3.6 ± 0.5 (N = 8 events from 4 cells). For AP triplets, detection accuracy was 89.3 ± 6.9% (N = 5 cells), and the SNR of the *detected* calcium transients was 5.6 ± 0.6 (N = 13 events from 5 cells). For trains of four APs, detection accuracy was 100.0 ± 0.0% (N = 3 cells), and the SNR of the calcium transients was 5.9 ± 0.7 (N = 5 events from 3 cells). When calculated on all calcium signals (*detected* and *undetected*) the SNR for single APs was 1.2 ± 0.1 (N = 63 events from 6 cells), for AP doublets the SNR was 3.0 ± 0.3 (N = 15 events from 4 cells) and for AP triplets the SNR was 5.5 ± 0.5 (N = 14 events from 5 cells).

The measured onset of the AP-induced calcium events (see Methods) was delayed by 8.4 ± 5.3 ms at 1 kHz (N = 46 events from 6 cells) and 17.0 ± 7.1 ms at 125 Hz (N = 61 events from 3 cells) compared to the onset of the AP train measured from juxtasomal recordings. These values represent the average temporal delay between the onset of the AP discharge and the onset of the detected calcium event. The error associated with these values can be taken as an estimate of the temporal precision in the identification of the spike or spike train onset. The plot of the temporal precision *vs* the SNR for all calcium signals recorded is shown in Supplementary Fig. S10. The dependence of the SNR on the acquisition frequency for single APs and train of APs is displayed in Supplementary Fig. S11. For experiments in Figs 1 and 4, we illuminated the cell with a single spot (laser power: 20–30 mW). Since the maximal laser power that we can achieve under the objective is ~300 mW, ~10–15 different cells can be simultaneously recorded under these experimental conditions.

### Simultaneous fast imaging and optogenetic inhibition

**Figure 5:**
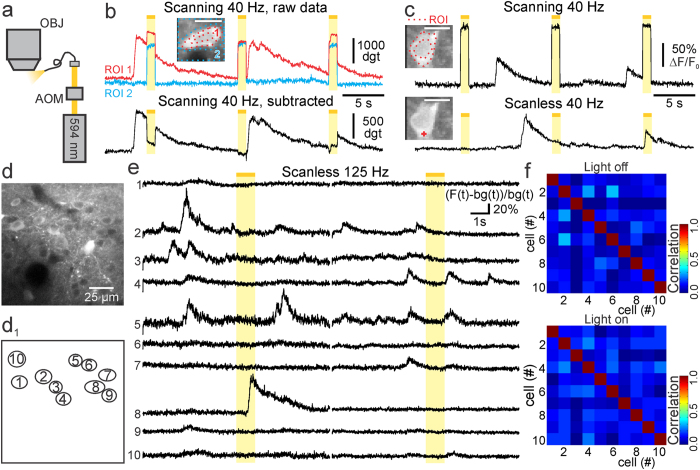
Reduced stimulation artifacts in scanless imaging. (**a**) Set-up for single-photon stimulation. Activation of Arch was performed at λ = 594 nm. Light was delivered through an optical fiber, and intensity was modulated with an acousto-optic modulator (AOM). OBJ: objective. (**b**) Scanning imaging of a GCaMP6s-expressing cell (inset) during single-photon stimulation (yellow bar) *in vivo*. Large artifacts are observed in cellular signals (red trace, ROI 1 in the inset) and in the surrounding region (blue trace, ROI 2 in the inset). Depth: 170 μm. Scales are expressed in digits (dgt; see Methods). (**c**) Scanning (top) and scanless (bottom) imaging of the same GCaMP6s-expressing cell (inset) during single-photon stimulation. Depth: 185 μm. Insets in b,c: scale bar 10 μm. (**d–d**_**1**_) Layer II/III neurons expressing GCaMP6s *in vivo*. Cells are numbered in (**d**_**1**_). (**e)** Simultaneous scanless imaging and single-photon stimulation in mice expressing GCaMP6s but no optogenetic actuator. Traces are expressed as (F(t) − bg(t))/bg(t). Depth: 175 μm. (**f**) Pair-wise correlation in the absence (light off, top) and presence (light on, bottom) of stimulation for the experiment displayed in (**e**).

We then coupled fluorescence imaging with optogenetic stimulation using wide-field single-photon illumination at λ = 594 nm (Fig. 5), the wavelength that is commonly used for activation of the inhibitory opsin Arch^19^. Visible light was delivered to the sample through an optical fiber (Fig. 5a), and experiments were initially performed in mice expressing only the fluorescent indicator and no optogenetic actuator. In the scanning configuration, large artifacts were observed in fluorescence signals during wide-field stimulation (Fig. 5b top). Importantly, background subtraction did not efficiently correct for these artifacts (Fig. 5b bottom). We compared the SNR of the stimulation artifact in the scanning and scanless configurations by recording from the same individual GCaMP6-expressing cells. We found that in scanless imaging artifacts were almost two orders of magnitude smaller than in the scanning mode (Fig. 5c), despite the fact that the same set of filters was placed in front of the camera and in front of the PMTs. The SNR of light-induced artifacts was 30.7 ± 2.0 in the scanning configuration and 0.4 ± 0.2 in the scanless configuration, p = 1.75E-22, Student’s *t*-test; scanning, N = 65 events from 10 cells; scanless, N = 69 events from 10 cells. This enormous reduction in optical artifacts is due to the different detection configurations (Supplementary Fig. S12). These experiments showed that, in the scanless configuration, the light-induced artifacts were greatly reduced and hardly detectable in individual cells. However, these data did not exclude the possibility that a small, almost undetectable artifact might be present in individual cells and might generate artificial correlation among pairs of cells during optogenetic stimulation when population imaging is performed. To test this possibility, we extended the previous results by recording in the scanless configuration from multiple neurons (Fig. 5d–f). In mice expressing GCaMP6s in layer II-III neurons and no inhibitory opsin, the average frequency of spontaneous calcium transients was not different in the absence and presence of yellow light illumination (1 s stimulus duration): average event frequency before stimulation, 0.08 ± 0.01 Hz; during stimulation, 0.08 ± 0.01 Hz (N = 123 cells from 15 experiments, paired Wilcoxon test, p = 0.75). Correlation of calcium events among cells was also unaltered by illumination with yellow light (Fig. 5f), confirming no effect of light stimulation on the detection of GCaMP6 signals in population imaging. The average network correlation was 0.28 ± 0.01 under control conditions (light off) and 0.28 ± 0.01 during optical stimulation (light on) (N = 15 experiments in 3 different animals, paired Student’s *t*-test, p = 0.33).

Because some inhibitory opsins can be excited through a two-photon absorption process^32^, using combined juxtasomal and scanless imaging experiments, we also controlled that projecting the near diffraction limited spot that is usually used to image GCaMP signals (power per point: 20–30 mW) directly on each recorded Arch-positive neuron did not affect the activity of Arch-expressing cells. To this aim, we recorded in anesthetized mice *in vivo* from mice expressing Arch and the red reporter tdTomato in parvalbumin-positive interneurons (Supplementary Fig. S13). Cytosolic tdTomato expression was used in these experiments to facilitate cell identification for juxtasomal recording.

**Figure 6:**
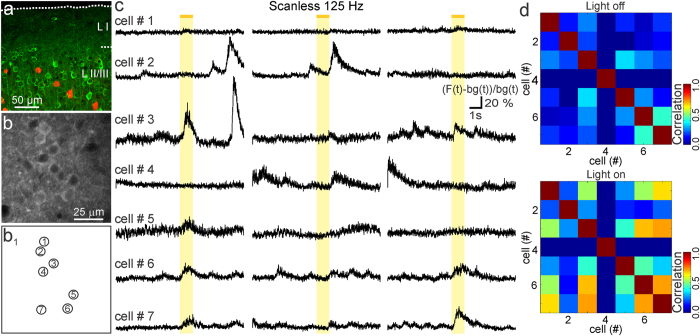
Inhibition of PV interneurons increases synchronous calcium signals in cortical neurons *in vivo*. (**a**) Confocal image showing GCaMP6s (green) in layer II/III neurons and tdTomato (red) expression in PV interneurons. (**b,b**_**1**_) GCaMP6s-expressing neurons. Cells are numbered in (**b**_**1**_). Depth: 190 μm. (**c**) Simultaneous scanless imaging and single-photon stimulation *in vivo*. Response to three light stimuli is shown. Traces are expressed as (F(t) − bg(t))/bg(t). (**d)** Pair-wise correlation in the absence (light off, top) and presence (light on, bottom) of wide-field stimulation for the experiment displayed in (**c**).

We finally combined fast scanless imaging with optogenetic inhibition and mapped with high spatial and temporal resolution the response of superficial cortical networks to the photoinhibition of a specific class of cortical interneurons, the PV-positive cells (Fig. 6). We injected AAVs transducing the GCaMP6s construct under the human synapsin-1 promoter in PV-Cre mice that were also injected with AAVs carrying a double floxed Arch construct. This combined injection resulted in the widespread expression of the calcium indicators in superficial cortical neurons and the selective expression of the floxed transgene in PV-positive interneurons (Supplementary Fig. S14, Fig. 6a). To avoid any potential effect of small light artifacts on pairwise correlations, fluorescence signals were calculated as (F(t)-bg(t))/bg(t) (see Methods and Supplementary Fig. S15). We found that during the optogenetic manipulation, GCaMP6-positive cells displayed enhanced calcium signaling (Fig. 6b–d). The frequency of calcium transients increased from 0.07 ± 0.01 Hz before illumination to 0.46 ± 0.03 Hz during illumination (N = 143 cells from 19 experiments, Wilcoxon test, p =7.5E-22). Not all cells responded to the optogenetic stimulation, and responding cells did not always respond to repetitive stimuli (Fig. 6c). Calcium events were more strongly temporally correlated among GCaMP6-expressing neurons during opsin stimulation than during the control periods (Fig. 6d): the average population correlation was 0.29 ± 0.02 under control conditions and 0.58 ± 0.04 during optogenetic suppression of the PV-positive neurons (paired Student’s *t*-test, p =1.0E-6, N = 19 experiments from 4 animals).

## Discussion

We developed a microscope based on patterned illumination using phase modulation that for the first time allows simultaneous, fast functional imaging and optogenetic photoinhibition in the intact mammalian brain. Two-photon imaging through patterned illumination has been previously applied to perform calcium imaging *in vitro*, in small organisms^21^,^22^,^33^,^34^,^35^,^36^ or in the mouse brain *in vivo*^37^,^38^. Here, our aim was to develop an efficient yet simple microscope design to achieve high-speed imaging of neuronal activity in the intact mouse brain while allowing simultaneous optogenetic manipulation of specific neuronal networks. When coupled with genetically encoded GCaMP6 indicators, this optical system allows mapping of suprathreshold activity of neurons with subcellular spatial resolution and high temporal resolution during the optogenetic manipulation. We validated this approach in anesthetized mice *in vivo* investigating the role of a specific class of interneurons, the PV-positive cells, in the modulation of the spatial and temporal profile of network dynamics in superficial cortical layers.

*In vivo* optogenetics is usually coupled with readout strategies that include intracellular and extracellular electrophysiological recordings^39^,^40^,^41^, functional magnetic resonance (fMR)^42^, positron emission tomography (PET)^43^, voltage-sensitive dye (VSD)^44^, intrinsic^45^ and calcium^46^ imaging. However, fMRI, PET, VSD and intrinsic imaging do not reach cellular resolution, while current *in vivo* electrophysiological approaches cannot record from the large numbers of closely-spaced individual cells that comprise local brain networks. In this respect, calcium imaging, in particular LSTPM, is an unique approach because it allows monitoring the activity of multiple nearby neurons within a given microcircuit, with subcellular resolution^2^. However, developing an all-optical system to couple fast two-photon calcium imaging with single-photon optogenetic manipulation *in vivo* is a challenging task for the reasons detailed below.

To date, the preferred solution to increase the acquisition speed in functional fluorescence imaging in the mammalian brain *in vivo* has been the use of random-access scanning microscopy using acousto-optic deflectors (AODs)^47^. Using this approach, the laser focus can be moved within microseconds from one location to another, according to arbitrary trajectories. Using an AOD-based two-photon microscope, 34 neurons could be imaged at 490 Hz in the intact mouse brain in 2D^9^ and 100 cells at 360 Hz in 3D^48^. However, being a sequential illumination scheme, the sampling rate depends on the number of imaged cells and on the number of illuminated points per cell needed to obtain a sufficiently high SNR^9^. Moreover, scanning imaging approaches, including fast AOD-based systems^9^,^10^, generally use PMTs as efficient fluorescence detectors. A crucial limitation of PMT detectors is that they are highly sensitive to the light intensities commonly used for optogenetic activation, preventing *simultaneous* imaging and optogenetic manipulation^16^,^17^. Finally, when coupling two-photon imaging of fluorescent calcium indicators with single-photon optogenetics, it must be considered that the visible light that is used for single-photon opsin excitation may interfere with the calcium indicator and that the infrared light that is used for imaging the indicator may result in opsin excitation^31^,^49^.

In combination with GCaMP indicators and Arch actuators, here, we developed a two-photon microscope based on wavefront engineering^50^ and camera detection that overcame these limitations, allowing fast (≥ 125 Hz) two-photon imaging during the optogenetic manipulation in living mice. Our approach is of crucial importance in optogenetic silencing experiments because the effects of inhibitory opsin activation on neuronal circuits are best observed during the optical stimulation.

In the scanless approach, the dwell time is independent of the number of imaged points within a FOV, and it can be as long as the inverse of the acquisition frequency (Supplementary Fig. S1b). Thus, in scanless imaging, the dwell time can be much longer than that in the scanning approach, leading to a larger number of emitted fluorescence photons at each ROI and, potentially, improved SNR. Combining imaging and juxtasomal electrophysiological recordings *in vivo*, we experimentally showed this (Fig. 1b,c) by comparing, at equal laser power, the SNR of calcium signals generated by single APs and trains of few APs in the same individual GCaMP6-expressing neurons under the two experimental configurations (scanning and scanless). Random-access two-photon imaging is currently considered the gold standard for high-speed two-photon imaging in the mouse brain *in vivo*^9^,^10^,^48^,^51^,^52^. Although random-access systems could *theoretically* scan ~16700^9^ or ~54300^10^ different positions in one second, thus reaching the performances achieved in the present study (i.e., 47 cells at 1 kHz), previous studies have not achieved these performances at extremely high acquisition rates (e.g., 1 kHz)^9^,^10^,^48^,^51^. In the present manuscript, we achieved this goal and demonstrated efficient recordings from neural networks comprising up to 47 cells at a 1 kHz acquisition frequency in the brain *in vivo*.

In addition to random-access microscopy, alternative approaches have been developed to increase the acquisition speed in functional fluorescence imaging, including, among others, light-sheet microscopy^53^, wide-field temporally focused excitation^54^, and light-field microscopy^55^. Light-sheet microscopy has been applied to large-scale functional imaging^56^,^57^,^58^,^59^ and rapid imaging of entire fixed mammalian brains^60^,^61^. Similarly, wide-field temporally focused^54^ and light-field^55^ imaging approaches have been used to perform brain-wide and whole-animal functional imaging, respectively. Parallel scanless approaches have also been developed to perform fast functional imaging, in particular in three dimensions. For example, combining structured illumination using wavefront engineering with volume projection imaging, it was possible to illuminate and collect light simultaneously from different focal planes both *in vitro* and *in vivo*^36^. Moreover, a hybrid design in which a conventional laser-scanning multiphoton microscope was combined with a 3D scanning-line temporal-focusing system was used to perform functional and structural volumetric imaging^62^. Many of the approaches described above may achieve and even surpass the speed performances of the system described in this manuscript. However, in contrast to the present work, most of these previous studies (but see ref. 56) were performed in rather transparent samples such as larval zebrafish^36^,^55^,^57^, *Caenorhabditis elegans*^54^,^55^, bioengineered neural tissue *in vitro*^62^, and mouse brain slices^36^, and many of these approaches still await validation in the highly scattering mammalian brain *in vivo*.

In our optical set-up, we developed a series of efficient solutions to decrease the light artifacts in fluorescence signals due to concurrent two-photon imaging and single-photon optogenetic stimulation. First, two emission filters were placed in series in front of the fluorescence detector. Although this may cause a small decrease in the collected fluorescence emission, it efficiently attenuated the contribution of backscattered single-photon light. Second, we used a camera as detector. This approach proved to be crucial for the successful combination of two-photon imaging and optogenetics. Indeed, the relative contribution of backscattered photons to calcium fluorescence signals is intrinsically reduced in the scanless compared to the scanning approach (Supplementary Fig. S12a,b), and experimental measures demonstrate that it is two orders of magnitude smaller in the scanless approach than in the scanning approach (Fig. 5c). Additional filters in front of the PMT would reduce backscattering of photostimulation photons in the scanning configuration, but they could also contribute to slightly affect the collection of fluorescence signals. One advantage of the scanless configuration is that it efficiently reduces the effect of backscattered photons limiting the need of additional filters.

In this study, we coupled two-photon imaging of GCaMP6 with single-photon excitation of Arch. This choice was dictated by the observation that GCaMP6 and Arch have rather well-separated single-photon absorption spectra^14^,^19^. However, due to the relatively high power that is used for optogenetic activation, we experimentally controlled for potential interference of single-photon light (λ = 594 nm) stimulation on GCaMP6 stimulation *in vivo*. In anesthetized mice, we verified that 594 nm light efficiently inhibited Arch-expressing neurons (Supplementary Fig. S13b–d, top panels) yet, when shined on mice expressing only the calcium indicator, it did not cause a significant change in spontaneous calcium events monitored with GCaMP6 and two-photon scanless illumination (Fig. 5c,e). This result suggests that our approach could also be used with red-shifted excitatory opsins^63^,^64^,^65^.

Using phase modulation of the laser beam, we restricted two-photon illumination (λ = 920 nm) only to those neurons that expressed the calcium indicators and not those expressing the inhibitory opsin Arch. However, even under these experimental conditions, we could not rule out the possibility that small processes of Arch-expressing cells could pass above or below the cell body of a GCaMP6-expressing cell and thus be exposed to two-photon illumination. Thus, to control for potential cross talk between light that is used for imaging on opsin activation, we showed that illumination with near-diffraction-limited spots that were delivered onto the cell bodies of Arch-expressing neurons did not modify the cells’ electrical activity (Supplementary Fig. S13a–d, bottom panels), raising the possibility that our method could also be extended to image cells in which the optogenetic actuator and the functional indicator are co-expressed in the same cells.

The scanless approach that we present in this manuscript has limitations. For example, this imaging modality requires splitting the laser beam into beamlets, each addressing a cell or region of interest. This may decrease the efficiency in fluorescent excitation but the extent to which this limits scanless imaging and which is the optimal number of beamlets to be used depends on a number of experimental parameters, including the total laser power that can be delivered to the sample and the fluorophore expression level. Under our experimental configuration, the total amount of available laser power under the objective was ~300 mW and we could image up to 47 different cells at 1 kHz, delivering <5.5 mW at each illumination site. Increasing the laser power available at the sample plane (improving for example the light transmission through the optical path) would allow imaging a larger number of cells or delivering more power at each illumination site, resulting in a more effective two-photon excitation. However, the application of high energy on biological samples may result in tissue heating and phototoxic effects. The optimal number of beams/scanning sites is thus dictated by the best trade-off between SNR and photodamage threshold and strongly depends upon experimental parameters that can be preparation or set-up specific. Improvements in the detection path, higher numerical aperture objectives, implementation of wavefront correction methods^66^,^67^,^68^ and the use of more efficient or spectrally shifted^69^ indicators may significantly increase the number of beamlets that can be efficiently used for scanless imaging. As for other imaging methods, motion artifacts may affect the quality of scanless imaging. In the present study, we performed analysis only in those scanless imaging sessions for which the comparison between two scanning images acquired just before and after scanless imaging reported no sign of movement. Moreover, we discarded those few scanless imaging sessions which showed clear signs of motion artifacts (in the form of fast and synchronous changes in fluorescence across all recorded cells). However, even by doing so we cannot exclude the presence of small artifacts that may be due to localized movements. Further improvements to address these issues may include, for example, the identification and illumination of specific regions of interest within the sample that are characterized by high contrast and that can be used as reference for the detection of local movement. The combination of patterned illumination with volume projection imaging may extend this methodology to 3D imaging^35^,^36^, while combining pattered two-photon stimulation of opsins^31^,^70^ with scanless imaging of calcium indicators may allow cellular resolution in optical manipulation during high-speed functional imaging. Furthermore, the scanless approach is sensitive to the scattering of emitted photons, limiting the applicability of this technique to the more superficial structures of the mammalian brain. However, our results demonstrate that it is possible to discriminate fluorescence signals coming from spots which are positioned ~10 μm apart in the x,y plane at 250 μm depth within the brain tissue with limited cross talk (Supplementary Figs S5 and 6), allowing the functional investigation of layers I and II of the mouse cortex and opening the possibility of applying this approach to gradient index- (GRIN) based endoscopes^71^.

In summary, our scanless patterned illumination approach allows for high-speed two-photon functional imaging during the optogenetic manipulation, even for extended durations of light stimuli. This greatly expands the range of applications and simplifies interpretation of the results of optogenetic manipulation, especially those involving inhibitory opsins. Moreover, reaching unprecedentedly high spatiotemporal resolution in fluorescence imaging during the optogenetic manipulation will enable the application of the scanless approach to dyes with faster kinetics than calcium sensors, such as voltage-sensitive fluorescence indicators^72^.

## Methods

### Animal surgery and viral injection

Experimental procedures involving animals have been approved by the IIT Animal Health Regulatory Committee and by the National Council on Animal Care of the Italian Ministry of Health (authorization # 34/2015-PR). All experiments were carried out according to the guidelines of the European Communities Council Directive. All animals were housed under a 12-hour light:dark cycle in individually ventilated cages, with a maximum of 5 animals per cage. Experiments were performed on young-adult (4–10 weeks old, either sex) C57BL/6 J mice (Charles River, Calco, Italy), PV-IRES-Cre mice (B6;129P2- *Pvalb*^*tm1(cre)Arbr/J*^, Jackson Laboratory, Bar Harbor, USA) and tdTomato reporter line animals (B6;129S6-*Gt(ROSA)26Sor*^*tm14(CAG-tdTomato)Hze/J*^, Jackson Laboratory, Bar Harbor, USA. The adeno-associated viruses (AAVs) AAV1.hSyn.GCaMP5G(GCamp3-T302L.R303P.D380Y). WPRE.SV40, AAV1.Syn.GCaMP6f.WPRE.SV40, AAV1.Syn.GCaMP6s.WPRE.SV40, AAV1.Syn.flex.GCaMP6s.WPRE.SV40, AAV1.CaMKII0.4.Cre.SV40, AAV1.CBA.Flex.Arch-GFP.WPRE.SV40, and AAV1.CAG.Flex.tdTomato.WPRE.bGH were purchased from the University of Pennsylvania Viral Vector Core. Viral injections in PV-Cre mice were performed on postnatal days 1–2 (P1–P2; the day of birth was designated as P0), while injections in C57BL/6 J wild-type mice were performed on juvenile (>P30) animals. Pups were deeply anaesthetized by hypothermia, and their heads were placed on a custom stereotaxic apparatus. The skull was exposed by a skin incision, and 200–300 nl of virus were injected at stereotaxic coordinates of 0 mm bregma, 1.5 mm lateral to the sagittal sinus, and 0.2 mm depth, by means of a glass micropipette. After the micropipette was removed, the skin was sutured. The pups were quickly revitalized under a heat lamp and subsequently returned to the dam. Adult animals were anesthetized with 2% isoflurane and placed into a stereotaxic apparatus (Stoelting Co, Wood Dale, IL). Mice were maintained on a warm platform at 37 °C during anesthesia. A small hole was drilled through the skull at stereotaxic coordinates 1.2 mm posterior to bregma and 2.5 mm lateral to the sagittal sinus. Over a period of 30 min, a volume of 0.5–1 μl was injected at depth of 300 μm. After surgery, the animals were positioned under a heat lamp and monitored until recovery. Two to four weeks after injection, mice were anesthetized with urethane (2 g/kg) or isoflurane (2%), and the skin above the skull was removed. A custom-made chamber with a central hole of 4 mm in diameter was attached to the animal’s skull to reduce motion-induced artifacts during imaging. A craniotomy (~ 300 × 300 μm^2^) was performed over the neocortex at least 500 μm away from the site of injection, and the dura was carefully removed. The surface of the brain was kept moist with normal HEPES-buffered artificial cerebrospinal fluid. Body temperature was maintained at 37 °C with a heating pad. Depth of anesthesia was assured by monitoring respiration rate, eyelid reflex, vibrissae movements, and reactions to pinching the tail and toe. In some experiments, oxygen saturation was controlled by a pulseoximeter (MouseOx, Starr Life Sciences Corp., Oakmont, PA). All incisions were infiltrated with lidocaine. For awake, head-restrained imaging experiments, a metal head plate was fixed to the skull via dental acrylic following the viral injection procedure. Animals were allowed to recover for one week before the training sessions began. For training, mice were habituated to the stereotaxic apparatus and the optical instrumentation for progressively increasing length of time (from 15 minutes to 1 hour) during ten consecutive days preceding the imaging session. At the end of the training period, mice were briefly anesthetized with isoflurane, and a small craniotomy was performed over the neocortex as described above. Animals were left to fully recover before starting calcium imaging recordings.

### Optical set up and imaging experiments

The microscope was composed of one pulsed Ti:Sapphire laser source (Ultra II Chameleon, Coherent, Milan, IT), a customized Prairie Ultima IV scan head (Bruker Corporation, former Prairie Technologies, Milan, IT), a liquid crystal spatial light modulator (SLM, X10468–07 SLM, Hamamatsu, Milan, IT) and an upright epifluorescence microscope (BX61 Olympus, Milan, IT). The laser beam intensity was modulated by a Pockels cell-based device and directed to the SLM by a series of mirrors (BB1-E03 Thorlabs, Newton, NJ). The SLM is a phase-only modulator for light linearly polarized in the direction corresponding to the liquid crystal orientation. Thus, a half-wave plate (λ/2 in Fig. 1a, RAC 5.2.10 achromatic λ/2 retarder - B. Halle Nachfl GMBH, Berlin, DE) was placed before the SLM. A first pair of lenses (L_1_ and L_2_ in Fig. 1a, AC254–030-B and AC254-075-B, respectively, Thorlabs; L_2_ was mounted on a SM1Z translation mount from Thorlabs) was used to resize the laser beam to fill the active window of the SLM. The SLM was mounted on a lab jack (L200/M, Thorlabs), a translator (PT1/M, Thorlabs), and a rotation platform (RP01/M, Thorlabs) to ensure necessary degrees of freedom for alignment. A second pair of lenses (L_3_ and L_4_ in Fig. 1a), AC254-300-B and AC254-150-B (Thorlabs), reshaped the laser beam to fit the dimensions of the scanning mirrors inside the scan head. All the mirrors used outside the scanhead were UM10-AG (Thorlabs). A third pair of lenses formed by the scan and tube lenses relayed the beam to the objective. A short-pass dichroic mirror (FF670-SDi01, Semrock Inc., Rochester, NY; *D*_*1*_ in Fig. 1a) reflected two-photon excitation light onto the sample and allowed the detection of emitted fluorescence (green line in Fig. 1a) via a camera (ORCA R2, Hamamatsu, Milan Italy) for experiments shown in Supplementary Fig. S3c–h and S5b, SciMeasure NeuroCCD-SMQ camera system (Redshirt Imaging, Decatur, GA) for experiments in other figures. In the NeuroCCD-SMQ camera, each image is divided in four quadrants corresponding to the four high performance very low noise output amplifiers^73^. To optimize fluorescence collection, an extra telescope (L_5_ and L_6_ in Fig. 1a, bottom, AC254-050-A and AC127-019-A from Thorlabs) was placed between D_1_ and the detector to match the FOV of the camera to the SLM addressable area at the sample when the SciMeasure camera was used. An IR-blocking filter (ET750SP-2P, Chroma Technology Corp, Bellows Falls, VR) and a specific emission filter (HQ525/50 M, Chroma Technology, Bellows Falls, VT) were positioned in front of the camera for fluorescence imaging. For simultaneous photostimulation and imaging experiments (Figs 5 and 6) a second emission filter (FF01-520/35, Semrock Inc, Rochester, NY) was added in front of the camera. For HQ525/50 M, T ~ 10^−7^ at 594 nm; T ~ 82% at 510 nm. For FF01-520/35, T ~ 10^−6^ at 594 nm; T ~ 97% at 510 nm. In the scanning configuration, the dichroic mirror D_2_ (Fig. 1a, top) was inserted in the emission path to deflect fluorescence signals to the photomultiplier tube (PMT). To compare the SNR of calcium events (Fig. 1b–d, Supplementary Fig. S2) and stimulation artifacts (Fig. 5c) in the two imaging configurations, experiments were performed on the same individual neurons alternating between scanning and scanless imaging sessions. For line scan experiments a ROI (1 × 64 pixels) was centered on the recorded cell and scanning was performed with 4 μs pixel dwell time at 0.98 ms/line. Importantly, the same set of filters was placed in front of the PMT (for scanning imaging) and the camera (for scanless imaging), and the same average laser power was used in the two recording configurations. Experiments presented in this study were performed using an Olympus LUMPlanFl40X/IR objective (0.8 NA), except those in Figs 2 and 3, in which we used an Olympus XLUMPlanFl20X objective (0.95 NA). At λ = 920 nm, the size of the field of view, defined as the maximal radial distance at which the spot intensity reaches 40% of the intensity of an equal spot positioned in the center of the FOV^74^, was ~115 μm with the 40X and ~200 μm with the 20X objective (Supplementary Fig. S4g). For scanless imaging, we first identified a number (*N*) of ROIs within the FOV by acquiring a high-resolution image in the scanning configuration. Using the SLM we then projected *N* spots, each targeted to a subregion of one ROI. Emitted fluorescence was detected in parallel with the camera. Because of limited diffraction efficiency, the effective intensity of the excitation spot decreased with the displacement from the center of the FOV. As a consequence, the intensity of the fluorescence emitted from ROIs positioned in the lateral part of the FOV was dimmer. We characterized the diffraction efficiency of our system (Supplementary Fig. S4g) by measuring the overall fluorescence intensity of a standard sample with different phase patterns applied to the SLM. We acquired images in the scanning configuration, while shifting the FOV with the SLM at different radial distances. The measurements has been performed at λ = 920 nm. To increase the SNR of fluorescence signals originating from lateral parts of the FOV, we used feedback software control of the SLM. We boosted the intensity of excitation light in lateral ROIs and partially compensated for the observed effect (see also below). In scanless imaging experiments in which multiple points were used, values of laser power per point were obtained by measuring the total power value under the microscope objective and dividing it by the number of projected points. Scanless imaging was always performed using diffraction-limited spots. Because of the undiffracted component and of the observed decrease in intensity in points projected to the distal part of the FOV, power values at the sample can be 20–30% smaller than the indicated number. To measure the PSF at different radial positions (Supplementary Fig. S4c,d), a single excitation spot (λ = 920 nm; microscope objective: LUMPlanFl40X/IR, 0.8 NA) was positioned over a subresolved fluorescent bead (0.17 μm in diameter) using phase modulation, and the PSF was acquired in scanning mode. To measure the PSF as a function of the number of simultaneously projected points, patterns of 1–20 points (randomly positioned within the FOV) were projected while one of the points was used to acquire the PSF of a subresolved fluorescent bead (0.17 μm in diameter).

The effect of scattering on fluorescence detection was quantified by imaging with the camera in the scanless configuration (excitation light, λ = 920 nm), either using a fluorescent bead (1.75 μm in diameter, Polyscience Inc., Warrington, PA) while interposing slices of cortical tissue of different thickness (100, 150 and 200 μm; 0 μm, no slice present) between the bead and the objective, or using *in vivo* imaging of the activity of cells located at different depths within the tissue. The ability of the scanless approach to separate signals coming from two adjacent neurons was evaluated by recording from two simultaneously active cells positioned ~10 μm apart at the positions *P1* and *P2* as a function of tissue depth. The intensity profile along a line connecting the two cells was then plotted, and the quantity *S* was defined as follows:


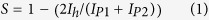


where *I*_*P1*_ and *I*_*P2*_ are the peaks in the intensity profile corresponding to the location of the two projected points (*P1* and *P2*), and *I*_*h*_ is the intensity value at a distance equal to half the distance between the two peaks. To evaluate the contamination of cell 1 on cell 2 we illuminated the two cells one at a time and calculating the quantity *C* = *I*_*c*_*/I*_*a*_, where *I*_*c*_ is the average intensity at cell 2 when cell 1 is illuminated and *I*_*a*_ is the average intensity of at cell 1 when cell 1 is illuminated.

Single-photon inhibition was performed by delivering 594 nm laser light (Mambo 100 mW, Cobolt AB, Solna, SE) through a multimode fiber (core diameter 200 μm, 0.22 NA, QMMJ-3X-UVVIS-200/240–0.4–6, OZ Optics Ldt, Ottawa, CA). Coupling between laser module and fiber was obtained via a 10X Olympus Objective (MPLN10X, Olympus, Milan, IT). Light-pulse control was performed via an acousto-optic modulator (R23080–3-LDT, Gooch & Housego PLC, Liminster, UK). The 594 nm light intensity was 15–35 mW at the fiber tip. The plaque that held the mouse’s head on the microscope stage was customized so that single-photon stimulation light did not result in a visual stimulus for the mouse.

### Phase modulation and software control

Custom code was written in LabView 2010 (National Instruments Corp, Austin, TX) to compute phase modulation maps, to manage the triggers (via PCI-6259, National Instruments) and to couple the SLM with the PrairieView software which controlled the laser scanning system. Excitation patterns with ensembles of points were generated by means of a Weighted Gerchberg-Saxton Iterative Fourier Transform Algorithm because of its superior illumination uniformity^75^. The custom code also controlled light intensity at each point, allowing the possibility to compensate for light loss at specific sites due to the presence of biological structures (e.g., blood vessels) above the ROI or to loss of efficiency in the distal part of the FOV. To compensate for these effects, we proceeded in the following way. We first created a phase map corresponding to *N* points. We then evaluated the fluorescence signal generated at every site (*I*_*m*_, with 1 ≤ *m *≤ *N*) and computed the average fluorescence (*Ī*) across sites. We defined ξ_*m*_as:


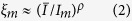


with *ρ* ∈ [0.5, 2] and ξ_*m*_ constrained in the range [0.5, 4]. The weight *w*_*m*_^75^ used to superimpose the hologram corresponding to the *m*^th^-site with the holograms of the other sites was then redefined:


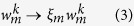


where ξ_*m*_ is fixed across the *k* algorithm iterations (with *k* ∈ ℕ), similarly to^71^. This procedure was iterated until the desired intensity at the site m-^th^ was achieved. A calibration routine with sub-micrometric precision was implemented to map the FOV obtained in scanning mode on the projection plane of the SLM at the sample. This procedure was based on a customized ImageJ plugin based on StackReg^76^ and through a TCP/IP communication protocol between the proprietary PrairieView software and the custom software. In imaging experiments, the field of interest was usually positioned such that the straight light of the zero-order component was projected to a region where non fluorescent structures were identified (e.g., the lumen of a blood vessel). Alternatively, the undiffracted component was shifted into an out-of-focus plane by moving the position of L_2_ (Fig. 1a) while keeping the modulated component in focus by imposing appropriate phase modulation on the SLM. When this latter solution was used, the same phase modulation applied to the SLM was also used in the laser-scanning configuration. For acquisition, each imaging session was organized in two steps. First, a high-resolution reference image of the sample was acquired in the scanning mode. Second, ROIs were identified, and based on the calibration routine and the software described above, a diffractive optical element was computed and superimposed onto the beam by the SLM to generate at the sample excitation profiles on the selected ROIs. Scanless imaging was then performed with a camera. After each scanless imaging session, a reference image of the FOV was acquired again in the scanning mode to control for potential movement artifacts.

### Two-photon targeted *in vivo* juxtasomal recording

Borosilicate glass patch pipettes (pipette resistance: 4–9 MΩ) were filled with ACSF solution mixed with Alexa Fluor 488 (20 μM). A craniotomy was opened in the mouse skull with procedures similar to those used for imaging (see above), and the patch pipette was lowered to the upper part of cortical layer II/III (120–150 μm from the brain surface). Neurons were targeted by imaging their fluorescence with the two-photon microscope while monitoring the pipette resistance by applying brief 5 mV voltage steps. When the pipette tip and the target cell were in close contact with each other, a negative pressure was applied to the pipette in order to achieve the juxtasomal configuration (resistance >20 MΩ). Electrical signals were amplified by a Multiclamp 700B, low-pass filtered at 10 kHz, digitized at 50 kHz with a Digidata 1440 and acquired with pClamp 10 (Axon instruments, Union City, CA). Electrophysiological traces were analyzed using Clampfit 10 software, and action potentials (APs) were detected according to a threshold criterion. Combined juxtasomal and imaging recordings in Fig. 1b,c and Supplementary Fig. S2a,b were conducted on the same individual cells, alternating between the scanning and scanless configurations.

### Image processing, data analysis and simulation

Temporal series recorded in the scanning and scanless configurations were imported into the open source ImageJ/Fiji software^76^ in order to identify ROIs. In scanless imaging, the fluorescence signal of a given ROI was measured by calculating the average intensity value in the four pixels closest to the illumination site within that ROI, and these signals were then analyzed with custom software. In line scan experiments the fluorescence signal was measured calculating the average intensity value in ~15–20 pixels located within the cell of interest. Fluorescence changes were computed as ΔF/F_0_ = (F(t) − F_0_)/F_0_, where F(t) is the fluorescence value at time t, and F_0_ is the fluorescence baseline which was calculated by first smoothing the trace with a moving average over 0.75 s and then calculating the minimal fluorescence level in a running window of ± 1.5 s (Fig. 2 and Supplementary Fig. S3c–j). In pharmacological and optogenetic experiments (Figs 3, 5e,f, 6), fluorescence changes were computed as ΔF/bg = (F(t) – bg(t))/bg(t), were F(t) is the average fluorescence value at time t in the four pixels closer to the illumination site and bg(t) is the fluorescence value of the background calculated at time t in an outer ring of twenty pixels (Supplementary Fig. S15a). In Fig. 5b, background subtraction was performed on raw data by subtracting point-to-point background fluorescence values (ROI 2, blue trace in the inset) from cell fluorescence values (ROI 1, red trace in the inset). When reporting raw data, fluorescent signals were reported in digits (dgt), indicating the digital pixel intensity value. The SNR for calcium events and artifacts during scanning and scanless illumination (Figs 1b, 1c, 5c, Supplementary Fig. S2 and Supplementary Fig. S11) was calculated as the ratio between ΔF and the standard deviation of the baseline noise. Baseline change was assessed on raw fluorescence traces by computing the quantity (B_f_ − B_0_)/B_0_, where B_f_ is the final baseline level, and B_0_ is the initial baseline value calculated in a window of ~0.5 s in the absence of activity. In experiments shown in Supplementary Fig. S3a–j and Fig. 3, to obtain a reliable and rhythmic fluorescence signal that could be used as reference, we boosted network activity by applying the GABA_A_ receptor antagonist gabazine (10 μM). The average integral of the fluorescence signal was calculated as:


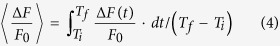


Correlation measurements were performed using calcium traces previously filtered with an exponential weighted moving average with a decay time τ = 15 ms. Next, the Pearson correlation between the calcium traces of every possible neuronal pair was computed. For the correlation matrix shown in Figs 2d and 3d, the correlation was evaluated over the entire duration of each recording and averaged across all recordings. In Figs 5f and 6d, the correlation was evaluated in a 1 s time window preceding the light stimulus (light off) and over the duration (1 s) of the light stimulus (light on). The average network correlation was calculated by averaging the correlation across all possible neuronal pairs. The instantaneous network correlation (panel c in Figs 2 and 3) was calculated by averaging correlations in overlapping sliding time windows of 1 s duration. To detect calcium events, traces were first filtered with an exponential weighted moving average with a decay time τ = 100 ms. The first derivative was then computed and averaged using a simple moving average in 100 ms time windows, similarly to a previous report^77^. A Gaussian fit centered at zero was used to extract the standard deviation (σ) of the noise of the processed signal. Transients exceeding the threshold of 4–5 σ were considered as calcium events with a refractory time of 150 ms between two consecutive calcium events. For the calcium traces acquired at 40 Hz (Figs 1b and 5b,c), the procedure was as described above, with the only exception that negative values of the processed signal were reset to zero. This operation allowed better identification of calcium events whose onsets occurred on the exponential decaying transients associated with previous calcium events. The time (*t*_1_) of the transients’ peak was used as an initial guess for estimating the onsets of the calcium events. The detection accuracy of action potentials was defined as the fraction of electrophysiological events that was associated with detectable calcium transients. The onsets of the detected calcium events were estimated based on the original calcium traces previously filtered with an exponential weighted moving average with a decay time τ = 5 ms. The calcium transients were fitted with the four-parameter function^9^:


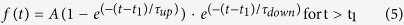



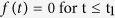


where all parameters were positive, and τ_up_ and τ_down_ were constrained to upper limits of 2 and 5 s, respectively. To calculate the precision of the calcium event onset in combined scanless imaging and juxtasomal recordings, *t*_1_ was compared to the time of occurrence of the first action potential discharged in the closest electrophysiological event, i.e., occurring within ± 100 ms. Electrophysiological events were separated into bursts of APs or single APs according to the following definition: a burst was identified when a train of APs was fired with an instantaneous inter-spike interval shorter than 100 ms; a single AP was not followed or preceded by any spike within 100 ms. Thus, the maximal burst frequency that could be detected was ≤10 Hz.

To estimate the contribution of the neuropil to the fluorescence signal generated by a point illuminating a target cell, we ran a simulation using a shell volume (green in Supplementary Fig. S7a) to represents the target GCaMP6-expressing neuron, and a virtually infinite volume to represent the neuropil outside the cell (light green in Supplementary Fig. S7a). The shell volume had external diameter 13.2 μm and thickness 3 μm. The dimensions of the shell were based on measurements from GCaMP6 expressing neurons under our experimental conditions. The surrounding volume had homogeneous fluorescence intensity that was set at 0.7 times the fluorescence intensity inside the shell, which is in accordance with^14^ and with our experimental observations. The FWHM values of the PSF (red oval in Supplementary Fig. S7a) were 0.7 × 0.7 × 2.7 μm^3^, similarly to the measurements reported in Supplementary Fig. S4c,d (obtained with an objective with NA = 0.8 at λ = 920 nm). Since in our scanless imaging configuration we delivered a single diffraction limited spot to the target cell, we first placed one diffraction limited excitation volume randomly inside the shell (excluding all points of the shell that were <0.5 μm from it outer limit), and we measured the fraction of fluorescence that was generated in the shell and the fraction that was generated in the outside volume. We then repeated this process for N iterations and computed the ratio (R) between the two fractions. R was 0.3 ± 0.1 (mean ± sd), N = 1000. This finding suggests that under our experimental conditions, ≤30% of the signal generated by one excitation spot may come from the neuropil outside the target region in accordance with previous calculations^78^.

We then estimated how much of the neuropil signal contaminating the fluorescence signal measured in cell1 also contaminates the fluorescence signal measured in cell2 because of the scattering of emitted photons. The fluorescence signals at the two locations (cell1 and cell2) were supposed of similar intensities. Based on the measurements displayed in Supplementary Fig. S6i, the fraction of signal generated at cell1 that contaminates the signal measured at cell 2 is <7% for tissue depth <250 μm. Thus, we concluded that the signal recorded at cell2 is only marginally contaminated by the neuropil signal excited at cell1.

Using a similar approach, we estimated how much the presence of a neighboring cell placed above or below the imaged cell could contribute to signal contamination. We first determined the distribution of the distance of one GCaMP6-positive cell from its first neighboring GCaMP6-expressing cell under our experimental conditions. Cell-to-cell distance was measured from the center of one cell to the center of the other cell. In our experiments the average radius of a cell was 6.6 ± 0.8 μm. Given the average cell dimensions (average diameter: 13.2 μm) and given the extension of the PSF along the axial direction (<3 μm) a diffraction limited spot positioned on cell 1 may cause significant excitation of fluorescence of a nearby cell 2 only if the cell-to-cell distance is below ~15 μm. Positioning the diffraction limited excitation spot for scanless imaging inside the shell (excluding all points of the shell that were <0.5 μm from it outer limit), we found that the relative signal contamination was <16% for cells that were 15 μm apart and that the contamination rapidly decreased with cell-to-cell distance. Importantly, only a minor portion of GCaMP6-labelled pairs (30 out of 643, <5%) have a cell-to-cell distance <15 μm, suggesting that, under our experimental conditions, this type of cross contamination occurs in a limited number of cases.

Given two cells in the scanless configuration, we evaluated whether period of high activity (i.e. discharge of multiple APs) in cell1 could be interpreted as calcium signal due to single AP discharge in an otherwise silent neighboring cell2. To this aim, we call ROI_1_ and ROI_2_ the regions of interest corresponding to cell1 and cell2. We then express the SNR in the two cells as:


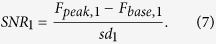


and:


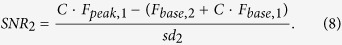


Where *SNR*_*1*_ and *SNR*_*2*_are the signal-to-noise ratios in ROI_1_ and ROI_2_, respectively; *F*_*peak,1*_ is the peak fluorescence signal in ROI_1_; *F*_*base,1*_and *F*_*base,2*_ are the baseline fluorescence in ROI_1_ and ROI_2_; *sd*_*1*_ and *sd*_*2*_ are the standard deviations of the fluorescence signal in ROI_1_ and ROI_2_.

Given that 0.01 < *C* < 0.07, then *C·F*_*base,1*_ ≪ *F*_*base,2*_. Moreover, if we approximate *F*_*base,2*_*≈ F*_*base,1*_ and *sd*_*2*_*≈ sd*_*1*_ then:


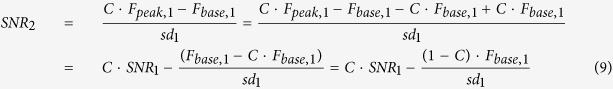


Given that:


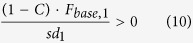


Then:


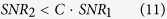


If cell 1 fires a train of 4 APs, *SNR*_*1*_ ~ 15 (see results section on page 10). From equation 11, we deduce that *SNR*_*2*_ < 1.1. Thus a train of APs in cell1 is unlikely to generate a signal high enough to be detected as a single AP in cell2.

### Immunohistochemistry

Animals were deeply anesthetized with urethane (2 g/kg) and transcardially perfused with 0.01 M PBS (pH 7.4) followed by 4% paraformaldehyde. Brains were post-fixed for 6 hours in the same solution, cryoprotected in a 30% sucrose solution in 0.1 M phosphate buffer (pH 7.4), and sectioned with a cryostat (Leica Microsystems, Milan, IT). Free-floating coronal sections (40 μm) were collected in multiwell dishes. For immunofluorescence, sections were incubated overnight at 4 °C in anti-GABA (1: 1000 rabbit, Sigma A2052) and anti-parvalbumin (1: 1000, mouse, Sigma P3088). Primary antibodies were diluted in 0.01 M PBS, pH 7.4, 0.5% Triton X-100, and 1% normal serum of the same species as the secondary antibody. Slices were then incubated for 1 hour at room temperature in goat anti-rabbit Alexa 647 (1: 800, Invitrogen A11034) or goat anti-mouse Alexa 647 (1: 800, Invitrogen A11029) secondary antibodies diluted in 0.01 M PBS, pH 7.4, 0.5% Triton X-100. Sections were then mounted, dried, and coverslipped with a DABCO [1,4-diazobicyclo-(2,2,2)octane]-based antifade mounting medium. Fluorescence images were acquired with a Leica SP5 inverted confocal microscope (Leica Microsystems, Milan, Italy).

### Statistics

Values are expressed as mean ± s.e.m unless otherwise stated. A Kolmogorov-Smirnov normality test was run on each experimental sample. When comparing two populations of data, Student’s *t*-test was used to calculate statistical significance in cases of Gaussian distribution; otherwise, the non-parametric Wilcoxon signed-rank (for paired comparison) test was used. All tests were two-tailed. When more than two populations of data were compared, one-way or two-way ANOVA with Bonferroni’s *post hoc* test was used in case of Gaussian distribution; otherwise, the non-parametric Kruskal-Wallis test was used. *p < 0.05; **p < 0.01; ***p < 0.001; NS, not significant.

## Additional Information

**How to cite this article:** Bovetti, S. *et al*. Simultaneous high-speed imaging and optogenetic inhibition in the intact mouse brain. *Sci. Rep.* **7**, 40041; doi: 10.1038/srep40041 (2017).

**Publisher's note:** Springer Nature remains neutral with regard to jurisdictional claims in published maps and institutional affiliations.

## Supplementary Material

Supplementary Figures
