# Corrigendum: Simultaneous high-speed imaging and optogenetic inhibition in the intact mouse brain

**DOI:** 10.1038/srep46122

**Published:** 2017-04-07

**Authors:** Serena Bovetti, Claudio Moretti, Stefano Zucca, Marco Dal Maschio, Paolo Bonifazi, Tommaso Fellin

Scientific Reports 7: Article number: 40041; 10.1038/srep40041 published online: 01 05 2017; updated: 04 07 2017.

The Acknowledgements section in this Article is incomplete.

“We thank J. Assad and M. Histed for comments on a previous version of the manuscript; V. Jayaraman, J. Akerboom, R. A. Kerr, D. S. Kim, L. L. Looger, K. Svoboda for GCaMP; and E. Boyden for Arch”.

should read:

“We thank J. Assad and M. Histed for comments on a previous version of the manuscript; V. Jayaraman, J. Akerboom, R. A. Kerr, D. S. Kim, L. L. Looger, K. Svoboda for GCaMP; and E. Boyden for Arch. This study was supported in part by grants from ERC (NEURO-PATTERNS), NIH Brain Initiative (1U01NS090576-01), EU FP7-Health-(DESIRE), San Paolo Foundation, MIUR FIRB (RBAP11X42L)”.

