# Supplementary material for: Simultaneous high-speed imaging and optogenetic inhibition in the intact mouse brain
Source: Sci Rep. 2017 Jan 5;7:40041. doi: 10.1038/srep40041 (PMC5215385; doi:10.1038/srep40041)
Supplement: Supplementary Figures [file srep40041-s1.pdf]

# Simultaneous high-speed imaging and optogenetic inhibition in the intact mouse brain

Serena Bovetti[1#], Claudio Moretti[1#], Stefano Zucca[1], Marco Dal Maschio[1], Paolo Bonifazi[2,3], Tommaso Fellin[1*]

[1] Optical Approaches to Brain Function Laboratory, Department of Neuroscience and Brain Technologies, Istituto Italiano di Tecnologia, Via Morego 30, 16163 Genova, Italy.

[2] School of Physics and Astronomy, Italy-Israel Joint Neuroscience Laboratory, Tel Aviv University, 69978 Tel Aviv, Israel.

[3] Computational Neuroimaging Lab, BioCruces Health Research Institute, Plaza de Cruces, s/n E-48903, Barakaldo, Spain

[#] Equal contribution

* Corresponding author

Supplementary figure S1

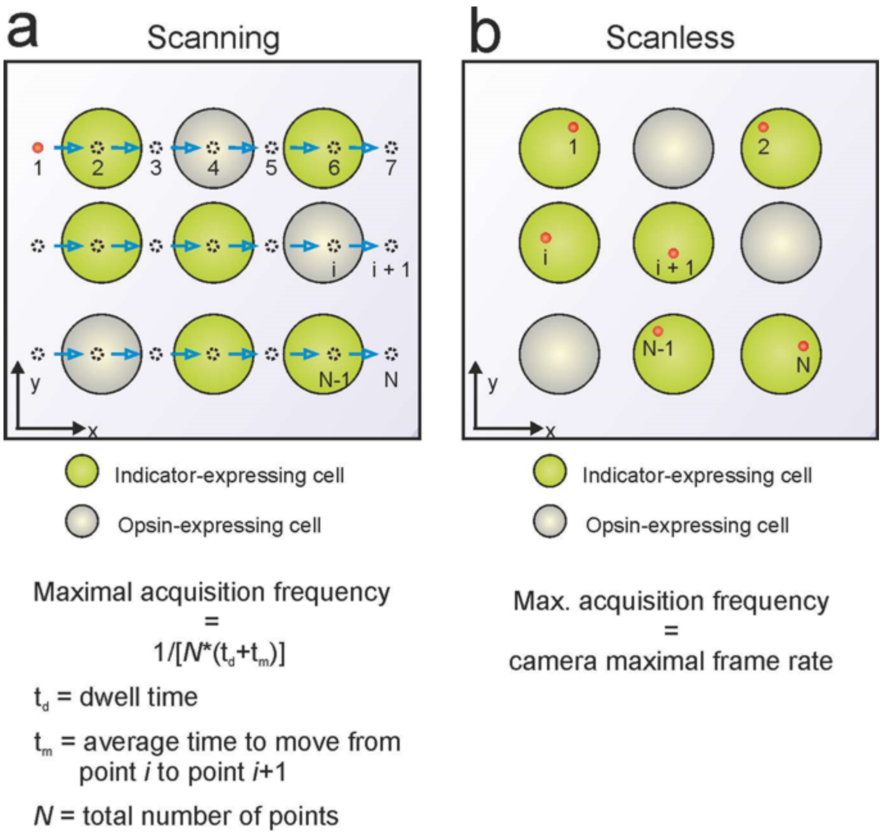


**Supplementary figure S1. Maximal acquisition frequency in scanning and scanless imaging. a**) In traditional raster scanning two-photon microscopes a spot (red dot) is sequentially deflected in raster modality through the whole field of view while emitted fluorescence is detected by a photomultiplier. In this configuration, the rate of image acquisition inversely depends on: *i)* the pixel dwell time ($t_d$), *ii)* the average time to move from one position to the other ($t_m$), and *iii)* the total number of points ($N$) that are sequentially illuminated at the sample. **b**) In the scanless configuration, all points of interest (red dots) are simultaneously illuminated. Emitted fluorescence signals are also simultaneously collected with a parallel detector, e.g., a camera. In this configuration, the maximal acquisition frequency of the system is a function of the maximal frame rate of the camera.

Supplementary figure S2

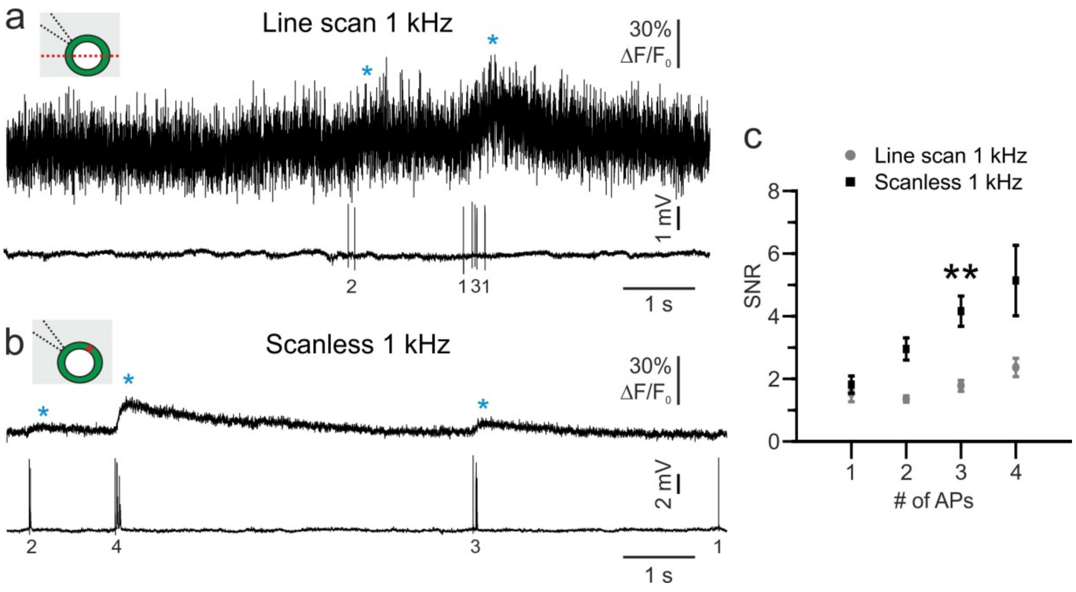


**Supplementary figure S2. SNR of GCaMP6 signals in the fast scanless configuration and in the fast line scan mode. a)** Simultaneous fast line scan imaging (top trace) and juxtasomal recording (bottom trace) from a GCaMP6s-expressing layer II/III neuron in an anesthetized mouse. Fluorescence signals from the juxtasomal recorded cell are imaged at 1 kHz acquisition frequency using 30 mW laser power. Number of discharged APs is indicated below the electrophysiological trace. The red dotted line in the inset indicates the trajectory of the line scan, the blue asterisks on the fluorescence trace indicate the detected calcium events. **b)** Simultaneous scanless imaging (top trace, acquisition frequency 1 kHz, laser power 30 mW) and juxtasomal recording (bottom trace) on the same cell imaged in line scan modality in (a). **c)** SNR as a function of the number of APs in the two recording modes. Comparison between imaging configurations was performed by monitoring only one cell (the juxtasomally-recorded neuron) in each imaging modality. Two-way ANOVA, p = 3E-4 for SNR *vs* imaging mode, p = 2E-2 for SNR *vs* AP number, interaction p = 0.29; 4-14 events from 6 cells.

Supplementary figure S3

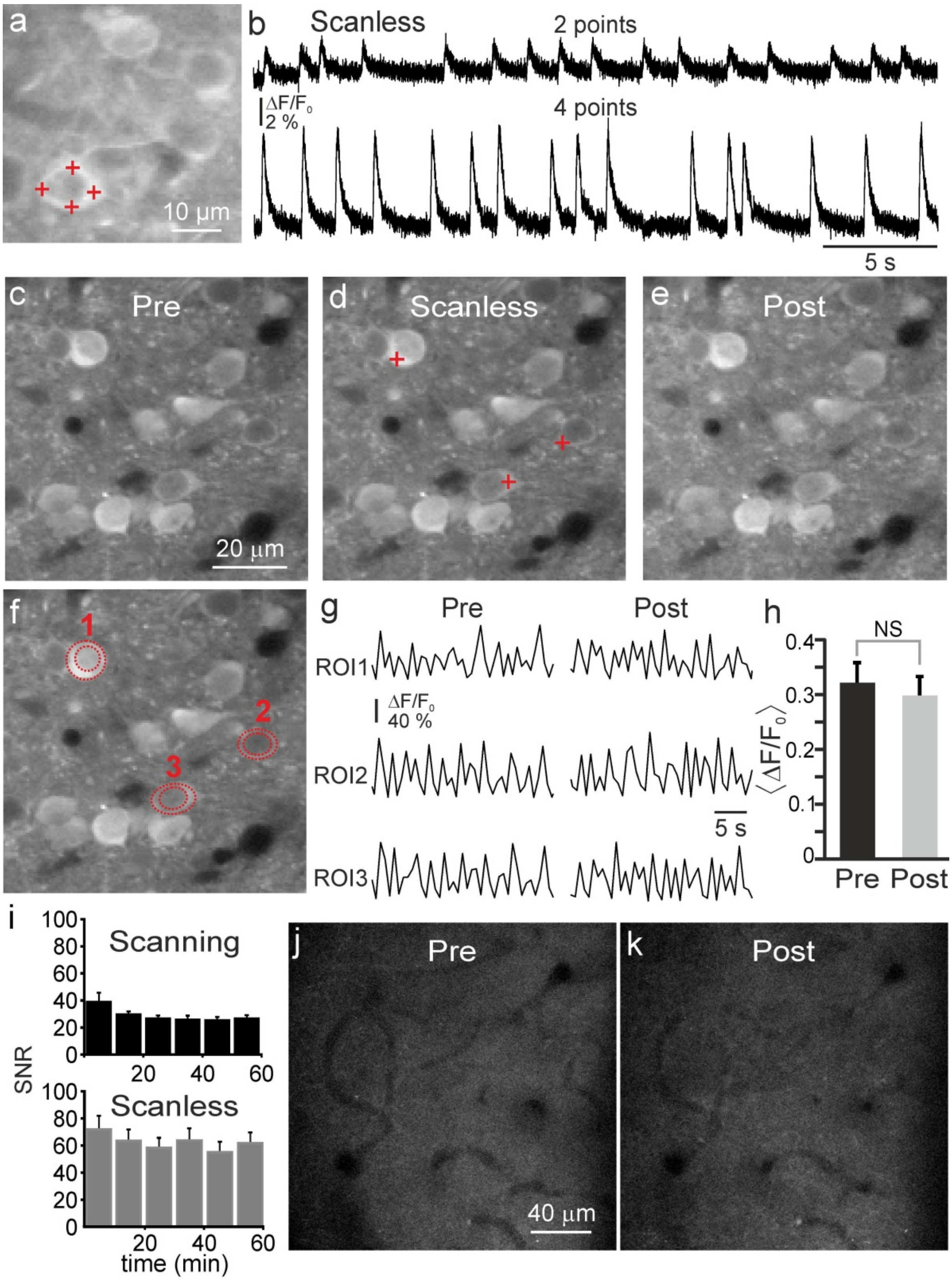


**Supplementary figure S3. Photobleaching and photodamage in scanless imaging. a)** GCaMP6-expressing cells in an anesthetized mouse. The red crosses indicate the positions of the spots of light that were used for imaging in the scanless configuration. **b)** Representative traces showing the fluorescence signals recorded in the scanless configuration illuminating one cell with 2 points (top trace) or four points (bottom trace). λ = 920 nm; power values 20 mW per spot. Depth: 200 μm. All

recordings displayed in this figure were performed in the presence of gabazine (10 μM). **c-e)** Scanning two-photon images of a FOV before (pre, c) and after (post, e) scanless imaging. Red crosses in (d) indicate the three positions that were simultaneously illuminated during scanless imaging (scanless). Laser intensity: < 30 mW per spot. **f-g)** Temporal series in the scanning mode before (Pre) and after (Post) scanless imaging were recorded. ROIs were positioned (red dotted lines in f) in the same cells in which scanless imaging was performed (red crosses in d). Representative traces of fluorescence signals are displayed in (g). **h)** Average values of integral of $\Delta F/F_0$ in cells recorded in the scanning mode before (Pre) and after (Post) scanless imaging. Paired Student's *t*-test p = 0.74, N = 11. **i)** SNR of fluorescence signals recorded in the scanning and scanless configurations over time. Laser intensity: 30 mW (scanning) and ~ 30 mW per spot (scanless). λ = 920 nm. Depth: 180 μm. Scanning: N = 14 cells from 5 animals, one-way ANOVA, p = 0.1. Scanless: N = 11-14 cells from 5 animals, one-way ANOVA, p = 0.8. **j-k)** Scanning two-photon images of the cortical surface above the illuminated area before (pre, j) and after (post, k) prolonged (1 hour) scanless imaging (3 minutes per recording session, one recording session every 10 minutes).

Supplementary figure S4

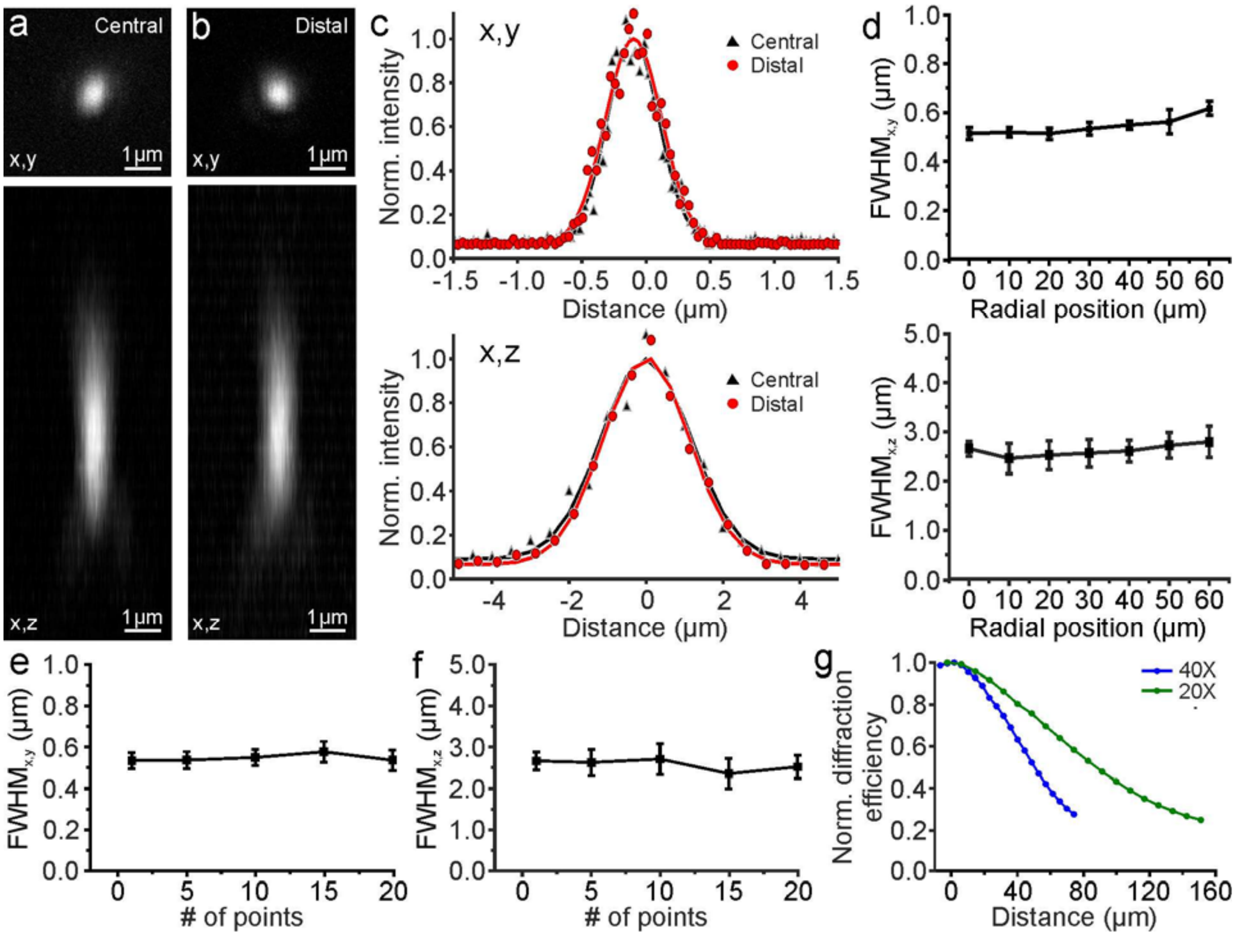


**Supplementary figure S4. Excitation point-spread function of the optical system. a-b**) x, y (top) and x, z (bottom) profiles of a fluorescent bead (0.17 μm in diameter) positioned at the center (a, central) and in the distal part (b, distal) of the FOV. The Olympus LUMPlanFl40X/IR, 0.8 NA objective was used. $\lambda$ = 920 nm. **c**) Intensity profiles of beads positioned at the center (black line) and in the distal part (60 μm from the center, red line) of the addressable FOV. **d)** Full-width-half-maximum (FWHM) values of the bead profile in the x, y plane ($FWHM_{x,y}$, top) and in the x, z plane ($FWHM_{x,z}$, bottom) as a function of the radial position. N = 8-12. Data in (d) are presented as mean ± standard deviation. **e)** Average values of $FWHM_{x,y}$ as a function of the number of simultaneously generated points that are projected on the sample. N = 8-12. **f)** Same as in (e) for $FWHM_{x,z}$. N = 8-12. Data in (e and f) are presented as mean ± standard deviation. **g)** Normalized diffraction efficiency as a function of the lateral position for the two objectives used in this study.

Supplementary figure S5

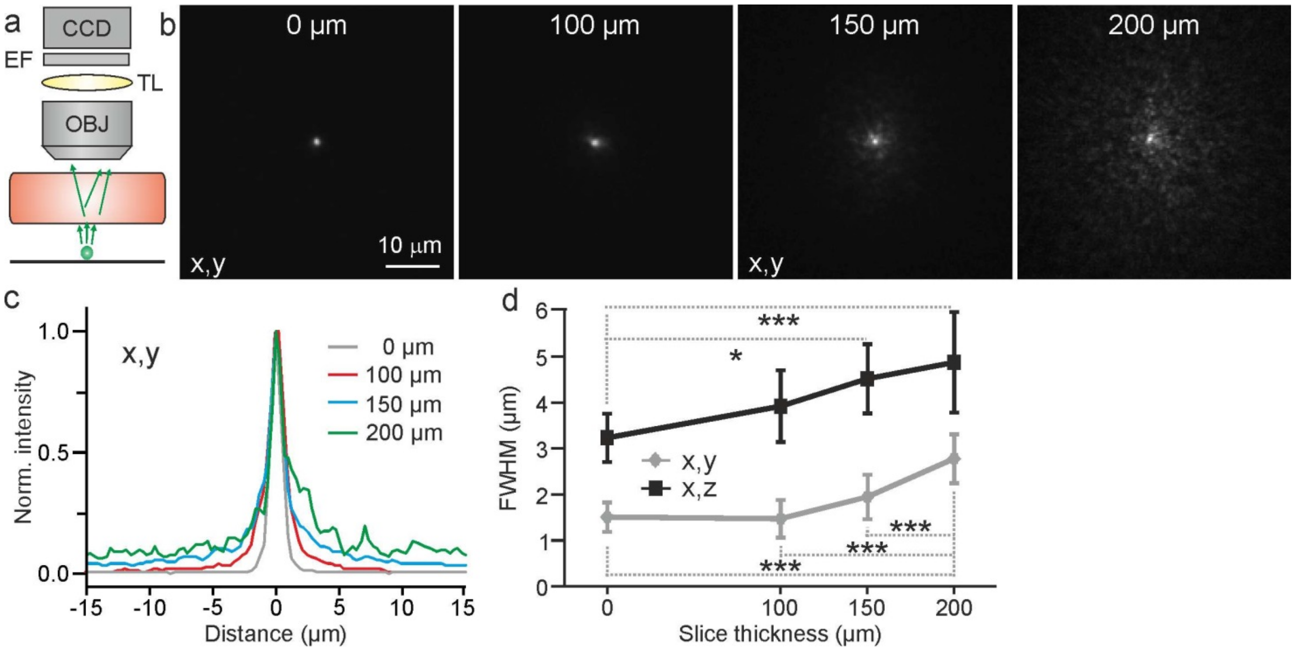


**Supplementary figure S5. Effect of tissue scattering on fluorescence imaging. a)** A fluorescent bead (1.75 µm in diameter, Polyscience Inc., Warrington, PA) was imaged in the scanless configuration (excitation light, $\lambda$ = 920 nm) with the camera while slices of cortical tissue of different thickness (100, 150 and 200 µm; 0 µm, no slice present) were interposed between the bead and the objective. **b)** Images of the bead in the x, y plane under the different experimental conditions. Scale bar applies to all images in (b). **c)** Intensity profiles along a line crossing the bead in the x, y plane for the different experimental conditions. **d)** Average FWHM values of the bead image in the x, y (grey) and x, z (black) planes under the different experimental conditions. One-way ANOVA, p = 4.3E-8 for FWHM values in the x, y plane and p = 7.9E-4 for FWHM values in the x, z plane, N = 8-13. Data in (d) are presented as mean ± standard deviation.

Supplementary figure S6

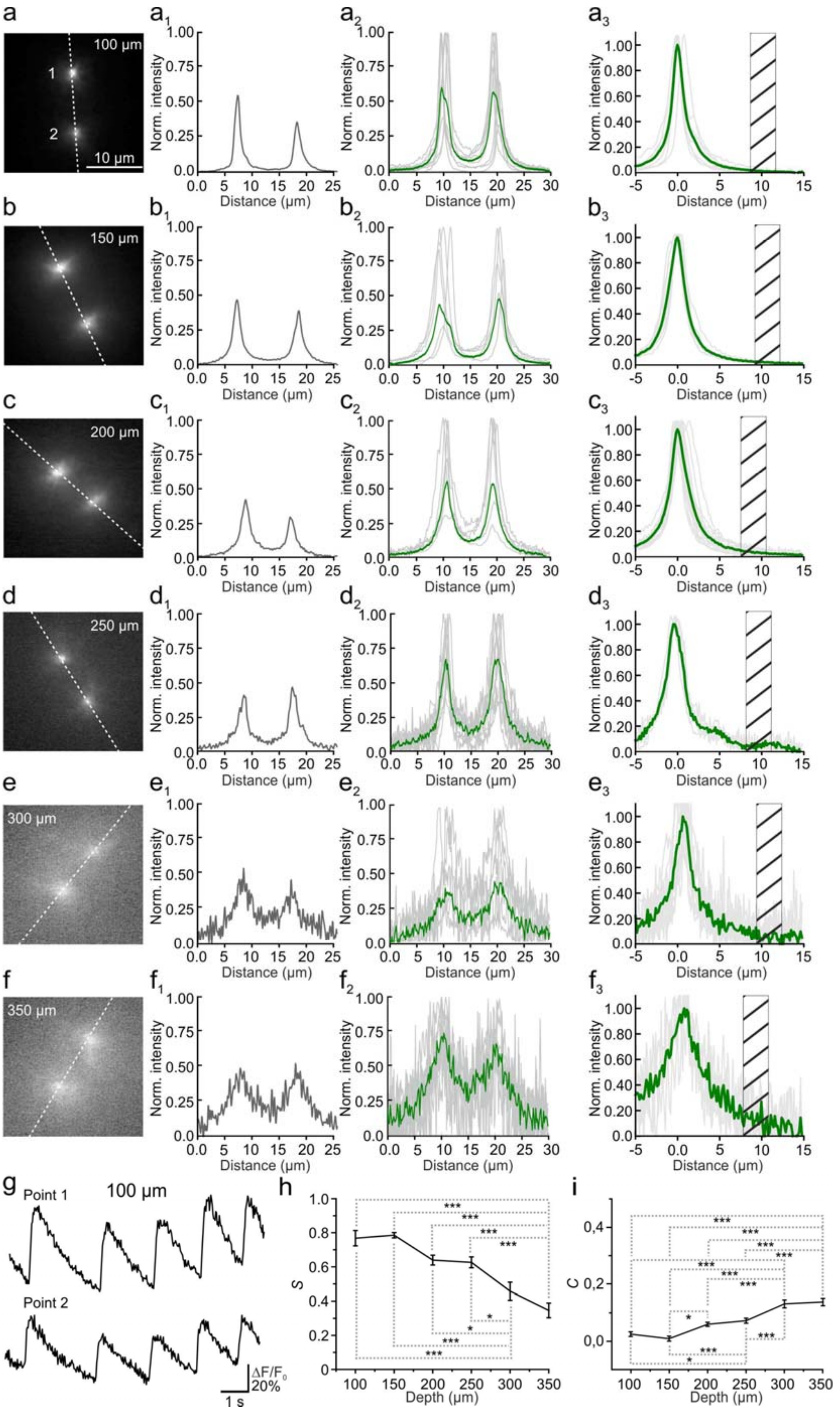

**Supplementary figure S6. Effect of scattering on fluorescence signals recorded from neighboring cells as a function of tissue depth. a-f)** Representative images of two neighboring (distance 8-12 μm) and simultaneously active cells located at different depths (100-350 μm) recorded in the scanless configuration in an anesthetized mouse. The cells expressed GCaMP6s and recordings were performed in the presence of the $GABA_A$ receptor antagonist gabazine (10 μM). Scale bar in (a) applies to b-f. **$a_1$-$f_1$)** Intensity profiles along the white dotted lines indicated in (a-f). **$a_2$-$f_2$)** The fluorescence profiles of all pairs of imaged cells (gray traces) are shown together with the average intensity profile (green trace) of all individual profiles. **$a_3$-$f_3$)** Fluorescence profiles of all pairs of imaged cells (gray traces) obtained illuminating only one of the two cells. The average intensity profile of all individual profiles is represented in green. **g)** Representative GCaMP6 signals from two simultaneously active cells located 100 μm deep within the cortex. Synchronous activity was elicited using the $GABA_A$ receptor antagonist gabazine. **h)** Ability to separate the peaks (*S*, see Methods) corresponding to two neighboring cells as a function of the neurons' depth within the tissue. One-way ANOVA, p = 5.05E-10, N = 7-10. **i)** Contamination (*C*, see Methods) of an active cell on a silent nearby ROI at different depths. One-way ANOVA, p = 2E-16, N = 14-20.

Supplementary figure S7

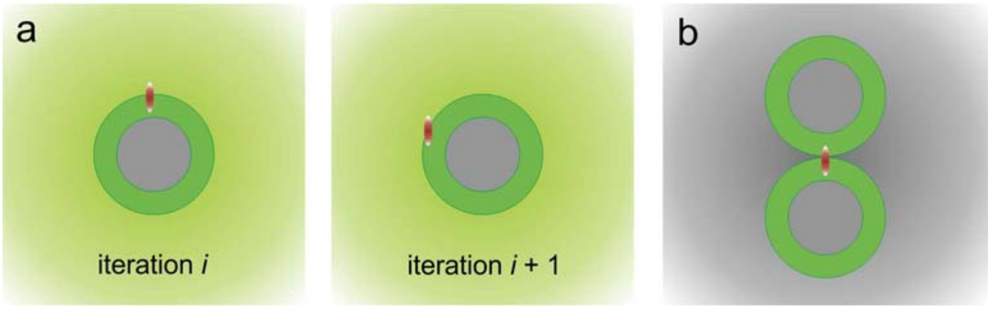


**Supplementary figure S7. Evaluation of neuropil contamination and cell-to-cell contamination. a)** The shell-like volume (green annulus shape in the image) represents a GCaMP6-expressing neuron, while the neuropil (light green) fills the space outside the cell. The excitation PSF (red oval) is placed within the green annulus shape in a diverse position at each iteration. **b)** Two neighboring cells (green annuli) are represented together with the excitation PSF (red oval). In this case the neuropil (grey) is considered not to contribute to the fluorescence signal generation.

Supplementary figure S8

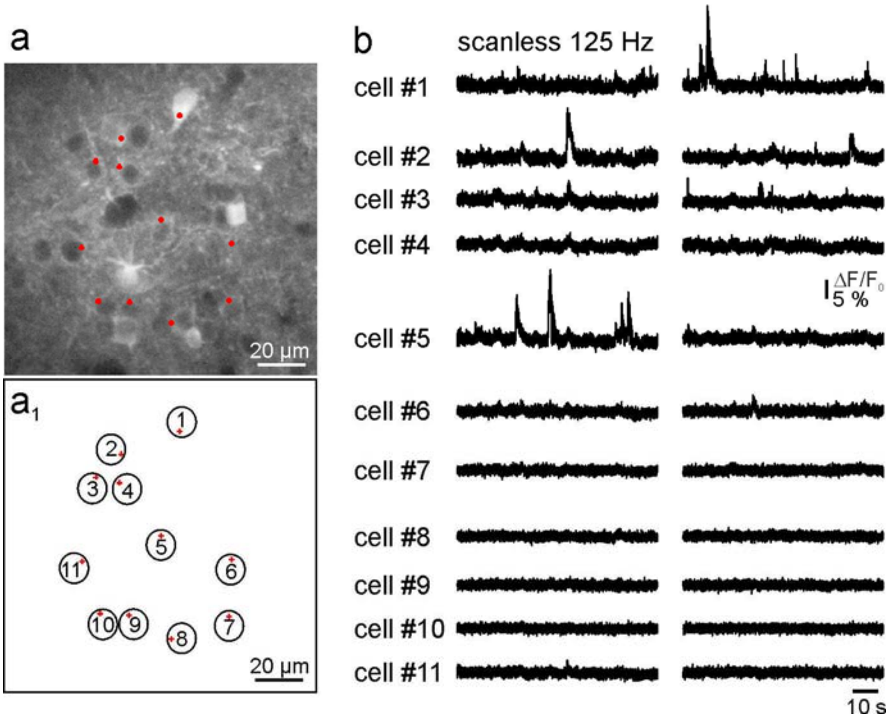


**Supplementary figure S8. Fast functional imaging of layer II/III cortical neurons in awake, head-restrained mice. a)** Scanning image showing layer II/III neurons expressing GCaMP6s in an awake, head-restrained mouse. The red crosses indicate the positions of the points that were projected for functional fluorescence imaging in the scanless configuration. **$a_1$)** Each cell is identified with a number. **b)** Fluorescence signals ($\Delta F/F_0$) over time from the 11 cells displayed in (a-$a_1$), imaged in the scanless configuration at 125 Hz. $\lambda$ = 920 nm; power, < 17 mW per spot. Depth: 163 µm.

Supplementary Fig. S9

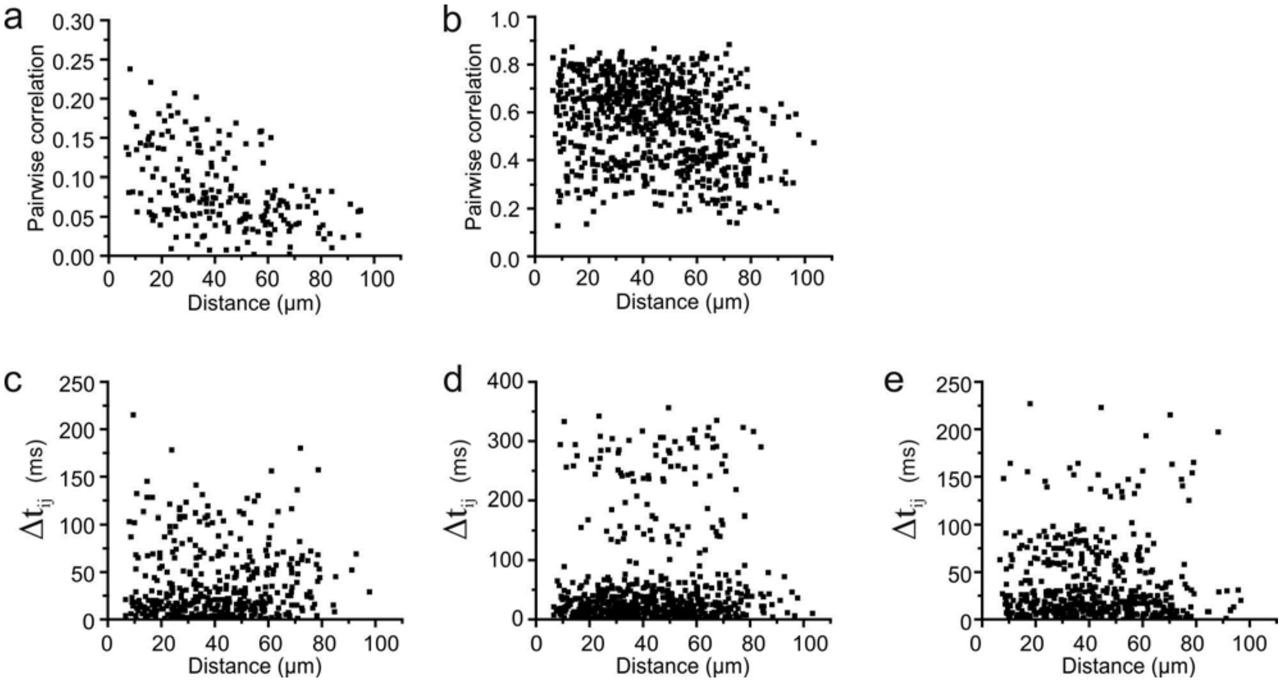


**Supplementary figure S9. Spatial organization of recorded network activities. a-b)** Pairwise correlation as a function of the distance between the two cells of the pair for the experiment shown in Fig. 2 (a) and Fig. 3 (b). **c-e)** Relative timing ($\Delta t_{ij}$) of calcium onsets between two cells as a function of the distance between the cells for the events shown in Fig. $3e_1$ (c), Fig. $3e_2$ (d), and Fig. $3e_3$ (e).

Supplementary figure S10

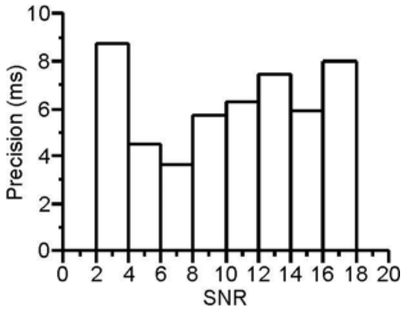


**Supplementary figure S10. Temporal precision *vs* SNR.** Temporal precision values as a function the SNR for calcium events (N = 259) recorded in the scanless configuration. Events with SNR < 2 were not included in this analysis.

Supplementary figure S11

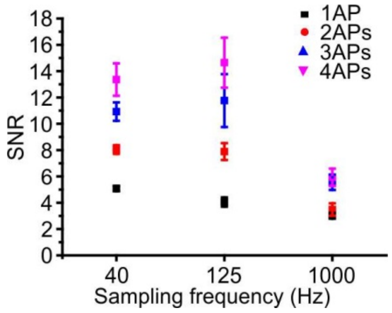


**Supplementary figure S11. SNR *vs* acquisition frequency.** SNR values of GCaMP6 signals as a function of the three acquisition frequencies (40 Hz, 125 Hz and 1 kHz) used in this study. Two-way ANOVA shows p = 3E-8 for SNR *vs* acquisition frequency, and p = 1E-12 for SNR *vs* AP number; interaction p = 0.24; N = 5-106 events.

Supplementary figure S12

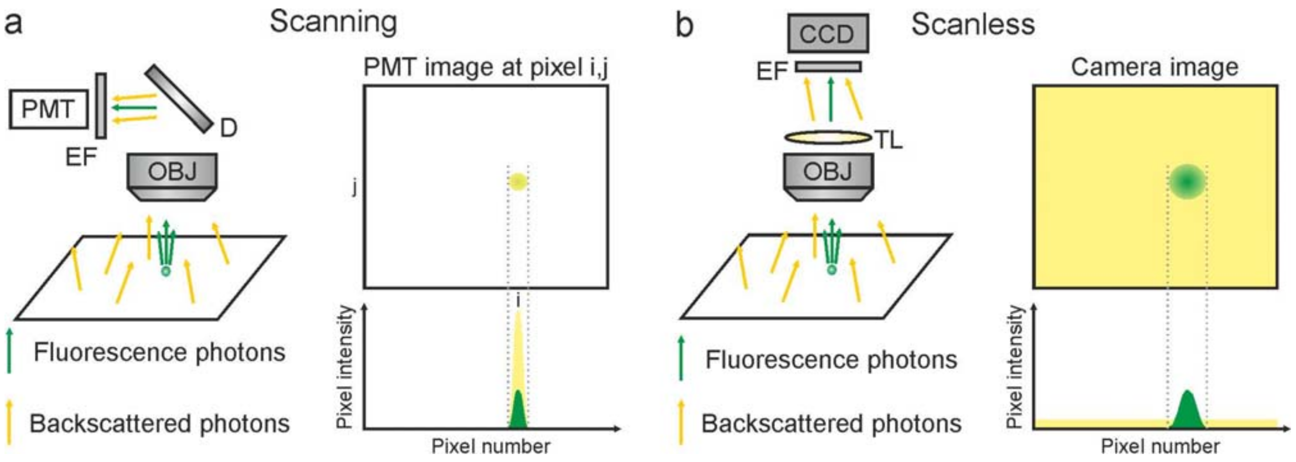


**Supplementary figure S12. Simultaneous two-photon fluorescence imaging and single-photon photostimulation in the scanning and scanless configurations. a-b)** Single-photon light that is used for optogenetic activation of Arch is backscattered by the brain and may enter the emission pathway. Because of the light intensities commonly used for Arch activation, a significant number of backscattered photons may reach the detectors despite high-optical-density emission filters. Backscattered photons are distributed across the whole FOV of the objective, but they contribute differently to the generation of the detector output signal in the scanning and scanless configurations. In the scanning configuration (a), backscattered photons arising from the whole FOV are spatially integrated and summed to the locally generated calcium fluorescence photons and all contribute to the PMT output current, which sets the intensity of a given pixel i,j. In contrast, in the scanless configuration (b), only a fraction of the backscattered photons (those reaching the detector in an area overlapping with that where fluorescence photons are distributed) are summed to the fluorescence ones. Thus, the relative contribution of backscattered photons to calcium fluorescence signals is intrinsically smaller in the scanless compared to the scanning approach.

Supplementary figure S13

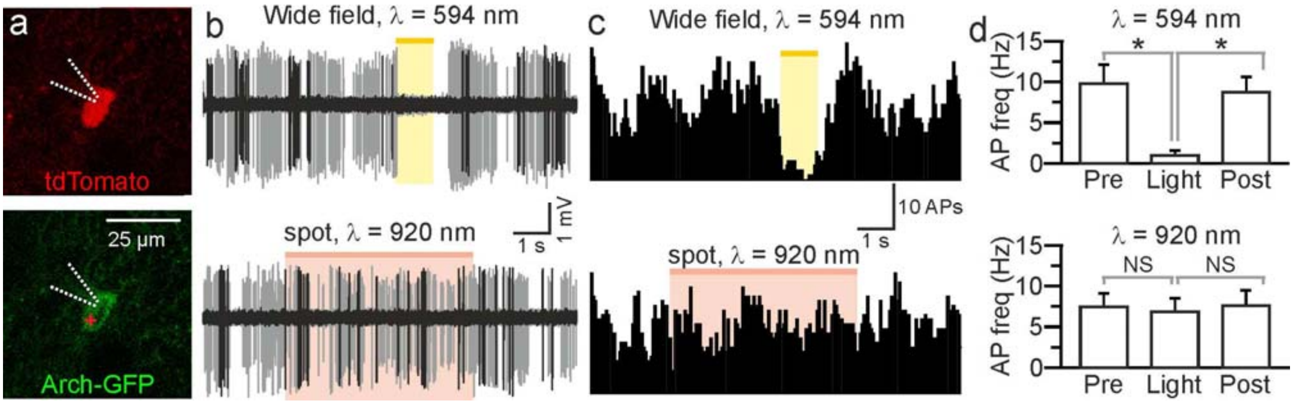


**Supplementary figure S13. No effect of scanless imaging on Arch-positive cells. a)** Two-photon image of a neuron expressing tdTomato (red, top panel) and Arch-GFP (green, bottom panel) *in vivo*. **b)** Juxtasomally recorded spikes from a tdTomato- and Arch-positive neuron *in vivo*. Seven consecutive sweeps are shown, with one sweep in black and the rest in grey. Illumination with a pulse of yellow light causes efficient suppression of AP firing (top traces). Illumination with a spot of infrared light (stimulus duration, 5 s; λ = 920 nm) positioned on the cell body (red cross in the bottom panel in a) has no effect on the cell's firing activity (bottom traces). Depth = 198 µm. **c)** Cumulative peristimulus time histograms showing the effect of wide-field yellow light illumination (top) and two-photon stimulation with a spot (bottom). Bin: 50 ms. **d)** Average values of AP frequency before, during and after wide-field illumination with visible light (top) and with spot illumination with infrared light (bottom). Top: one-way ANOVA, $p = 8E-3$, $N = 5$ cells from 3 animals. Bottom: one-way ANOVA, $p = 0.96$, $N = 5$ cells from 3 animals.

Supplementary figure S14

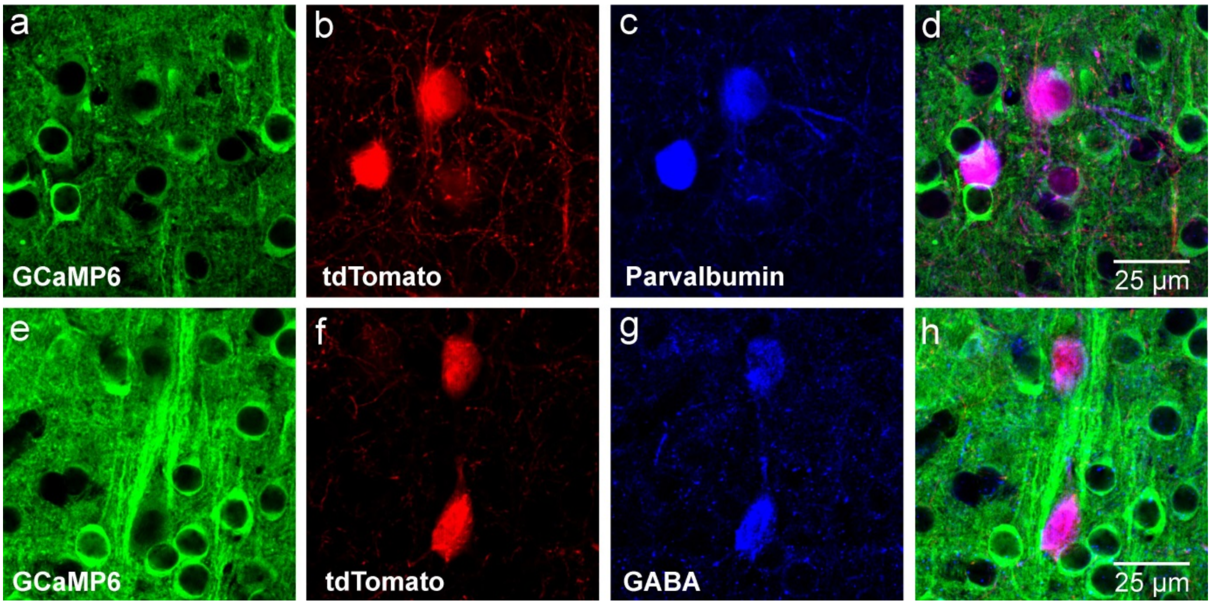


**Supplementary figure S14. Transgene expression in PV-positive interneurons. a)** Confocal image showing GCaMP6s expression in layer II/III neurons, which was obtained through the injection of AAVs carrying the hSynapsin1-GCaMP6s construct into PV-Cre mice. **b)** Same field as in (a), showing tdTomato expression in PV interneurons. Expression of tdTomato in PV cells was obtained injecting PV-Cre mice with AAVs carrying the Cre-dependent tdTomato sequence. **c-d)** Confocal image showing parvalbumin staining (c). Images in (a-c) are shown merged in (d). **e-h)** Confocal images showing GCaMP6 (e), tdTomato (f) and GABA staining (g) in sections from PV-Cre mice co-injected with AAVs carrying an hSynapsin1-GCaMP6 construct and AAVs carrying the Cre-dependent tdTomato sequence. Images in e-g are shown merged in (h).

Supplementary figure S15

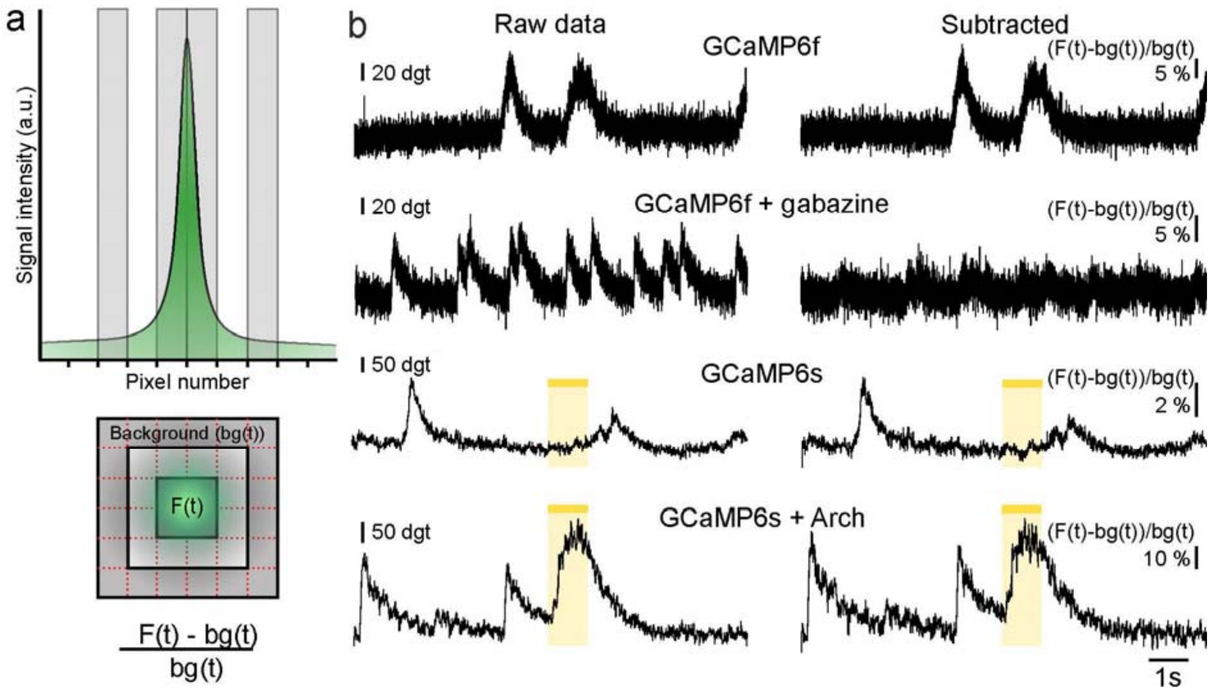


**Supplementary figure S15. Analysis of calcium signals recorded in the scanless configuration.** **a)** Schematic showing the criterion that can be used to analyze calcium signals in the scanless configuration. **b)** Left: raw fluorescence signals recorded in the scanless configuration during spontaneous cortical activity in mice expressing GCaMP6f in the absence of gabazine (GCaMP6f, see also Fig. 2), in the presence of gabazine (GCaMP6f + gabazine, see also Fig. 3), during simultaneous scanless two-photon imaging and single-photon photo-stimulation in mice expressing only GCaMP6s (GCaMP6s, see also Fig. 5), and during simultaneous scanless two-photon imaging and single-photon photo-stimulation in mice expressing GCaMP6s and Arch (GCaMP6s + Arch, see also Fig. 6). Scales for raw data are expressed in digits (dgt, see Methods). Right: the method displayed in (a) was applied to the raw traces shown in the left panel in (b).
